# Omics exploration of deep-sea biodiversity: data from the “*Pourquoi Pas les Abysses?*” and *eDNAbyss* projects

**DOI:** 10.1038/s41597-025-06009-1

**Published:** 2025-12-20

**Authors:** Sophie Arnaud-Haond, Blandine Trouche, Cathy Liautard-Haag, Karine Alain, Johanne Aubé, François Bonhomme, Miriam I. Brandt, Annaëlle Caillarec-Joly, Marie-Anne Cambon, Florence Cornette, Valérie Cueff-Gauchard, Patrick Durand, Colomban de Vargas, Christine Felix, Sandra Fuchs, Genoscope Technical Team, Babett Günther, Nicolas Henry, Stéphane Hourdez, Didier Jollivet, Anne-Sophie Le Port, Françoise Lesongeur, Loïs Maignien, Sophie Comtet-Marre, Marjolaine Matabos, Emmanuelle Omnes, Pierre Peyret, Florence Pradillon, Jozée Sarrazin, Clemens Schauberger, Adrien Tran Lu Y, Osvaldo Ulloa, Sandrine Vaz, Daniela Zeppili, Frédérique Viard, Frédérick Gavory, Shahinaz Gaz, Julie Guy, E’Krame Jacoby, Pedro H. Oliveira, Gaëlle Samson, Jean-Marc Aury, Patrick Wincker, Stéphane Pesant, Julie Poulain, Caroline Belser

**Affiliations:** 1 MARBEC, Univ Montpellier, IFREMER, IRD, CNRS, Sète, France; 2 Univ Brest, Ifremer, BEEP, F-29280 Plouzané, France; 3 ISEM, Univ Montpellier, CNRS, IRD, Montpellier, France; 4 NORCE, Norwegian Research Centre AS, Climate & Environment department, Bergen, Norway; 5 Ifremer, IRSI, SeBiMER Service de Bioinformatique de l’Ifremer, Plouzané, France; 6 Sorbonne Université, CNRS, Station Biologique de Roscoff, UMR 7144 Adaptation and diversity in the marine environment, Place Georges Teissier, Roscoff, France; 7 Research Federation for the Study of Global Ocean Systems Ecology and Evolution, FR2022 GOSEE, 3 rue Michel-Ange, Paris, 75016 France; 8 CNRS, Sorbonne Université, FR2424, ABiMS, Station Biologique de Roscoff, Roscoff, 29680 France; 9 UMR 8222 LECOB CNRS-Sorbonne Université, Observatoire Océanologique de Banyuls, Avenue du Fontaulé, 66650 Banyuls-sur-mer, France; 10 Université Clermont Auvergne, INRAE, UMR 0454 MEDIS, Clermont-Ferrand, France; 11 Hadal & Nordcee, Department of Biology, University of Southern Denmark, Odense, Denmark; 12 Instituto Milenio de Oceanografía (IMO), Universidad de Concepción, Concepción, Chile; 13 Génomique Métabolique, Genoscope, Institut François Jacob, CEA, CNRS, Univ Evry, Université Paris-Saclay, Evry, France; 14 Genoscope, Institut François Jacob, Commissariat à l’Energie Atomique (CEA), Université Paris-Saclay, 2 Rue Gaston Crémieux, Evry, France; 15 European Molecular Biology Laboratory, European Bioinformatics Institute, Wellcome Genome Campus, Hinxton, Cambridge, CB10 1SD UK; 16 Present Address: GEOMAR, Helmholtz Centre for Ocean Research Kiel, Wischhofstraße 1-3, 24148 Kiel, Germany

**Keywords:** Biodiversity, Marine biology, Community ecology

## Abstract

The deep-sea floor encompasses more than half of the surface of our planet, yet the extent and distribution of deep-sea biodiversity and its contribution to large biogeochemical cycles remain poorly understood. This knowledge gap stems from several factors, including sampling issues, the magnitude of the work required for morphological inventories, and the difficulty of integrating results from disparate local studies. The application of meta-omics to environmental DNA now makes it possible to assemble interoperable datasets at different spatial scales to move towards a global assessment of deep-sea biodiversity. We present a large-scale dataset on deep-sea biodiversity, with data and metadata openly accessible at ENA and Zenodo. The resource was generated using standardized protocols developed according to FAIR principles, covering fieldwork through bioinformatic analysis, within “*Pourquoi Pas les Abysses?*” and *eDNAbyss* projects. Together with information ensuring reproducibility, this dataset —combining metagenomics, metabarcoding across the Tree of Life and capture-by-hybridization— contributes to the international concerted effort to achieve a holistic view of the biodiversity in the largest biome on Earth.

## Background & Summary

The ocean covers approximately 70% of our planet, two-thirds of which lie below 3500 metres in depth and shelter still unexplored areas of Earth biodiversity^1^. The deep sea encompasses a broad genomic repertoire and possibly the largest archive of information about the evolution of life on Earth. It also contributes to a vast number of realized or potential services but is subject to increasing anthropogenic impacts^2,3^. Classical biodiversity studies based on morphological recognition and the description of taxa (some of which started in the late 19^th^ century) allowed researchers to scratch the surface of this vast biome, showing that the low biomass characterizing the abyssal plains is coupled with a high level of local diversity and heterogeneity^4–6^. Nearly all Animalia phyla (27 of the 31 recognized by the world register of marine species Worms^7^) were found in the deep sea with, as of January 2025, slightly more than 30,000 species listed in the “World Register of Deep Sea Species” (WoRDSS^8^). While the first studies focused on abyssal plains and canyons, the discovery of chemosynthetic ecosystems in the late 1970s expanded the scope of deep-sea research and reinforced the view that the deep sea is a rich source of biodiversity distributed in diverse distinct ecosystems^1,9–11^. Abyssal plains and hills represent more than half of deep seascapes, while the geomorphological features of the deep ocean^12^, where many new organisms are being discovered, are the least explored, revealing distribution heterogeneities driven by several environmental parameters operating at different spatial scales^13–15^. Since the 10 million species proposed by Grassle and Macjoleck^6^, large uncertainties and vivid debates have characterized our appraisal of the number of deep-sea species (and possibly ecosystems) still to be discovered^16–18^ in this largely undersampled part of Earth^19^. Some authors suggest that deep-sea biodiversity may exceed that present in coastal areas^20^, whereas others consider that the imbalance between biodiversity in the deep sea and that in shallow water is well documented^17^. The diversity of deep-sea species that contribute to essential biogeochemical cycles (carbon, sulfur, etc.), water column processes, the transport of primary and secondary products^18,21^, their interactions and the drivers of their distributions remain largely unknown. This lack of knowledge hampers the understanding of their precise roles in the ecology of the marine environment as a whole and is of concern for the conservation of this still enigmatic compartment, particularly in view of increasing anthropogenic pressures on the deep sea, including increasing prospects for deep-sea mining^3^.

The synthesis of results obtained across various local and regional studies led us to propose hypotheses regarding the influence of historical and contemporary environmental parameters (e.g., depth, latitude, sediment properties, deep water mass properties, concentration of particulate organic carbon [POC] sinking from the surface, etc.) shaping the nature and distribution of biodiversity on the seafloor^17,22^. These interpretations were admittedly limited by i) the vastness of the task, involving metre squares of sediments/rocks and countless hours in the laboratory to sort, recognize or describe species, having limited the studies to local, and at best regional scales; ii) the lack of standardization of sampling gear and sampling protocols (not always realistic in the deep sea beyond a certain level of complexity), preventing comparisons or integrated inventories (among others owing to observer effects linked to species description and identifications^23–25^); and iii) the microscopic nature of many organisms severely limiting the global inventory of life in the deep sea and the understanding of the contribution of this vast biome to global Earth biological diversity and functioning. In recent years, the exploitation of environmental DNA and high-throughput sequencing has provided snapshots of diversity and confirmed the extent of biodiversity and taxonomic gaps in the deep sea^26,27^; however, the use of a panel of distinct sampling and molecular protocols prevents the full integration of studies to extract global patterns across the tree of life. Several worldwide projects have led to global-scale analyses of the microscopic world in the water column (OSD, Tara^28–30^, Malaspina^31^), yet no such project has yet taken place to integrate deep-sea benthos.

**Fig. 1:**
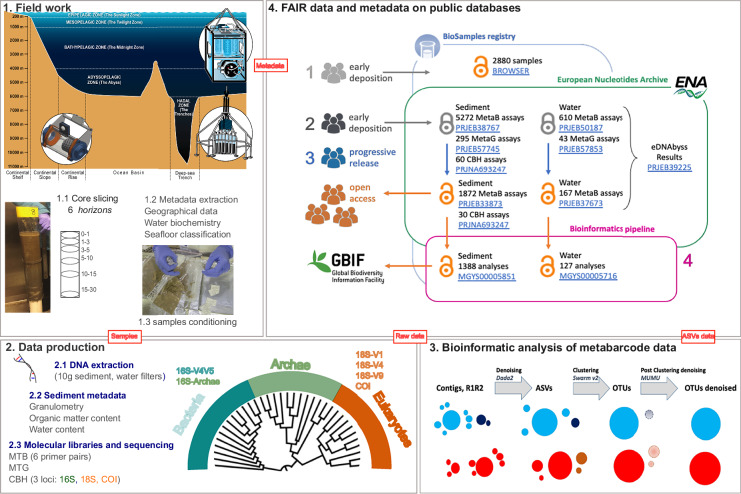
Visual summary of the eDNAbyss workflow, from sample to open data. Field work in marine realms through the water column and sampling gear favoured the sampling of eDNA to reveal deep-sea biodiversity. High-volume “SALSA” pump (upper right) systems, ELFES hard substratum (lower left) and multicore sediment sampler (lower right) (© Pierre Lopez), 2. Data production for metagenomics, metabarcoding and capture; 3. Bioinformatic analysis of metabarcoding data, and 4. FAIR data and metadata on public databases with details of the already available data in open access as of May 2025 and the accession numbers of bioprojects in blue.

**Fig. 2:**
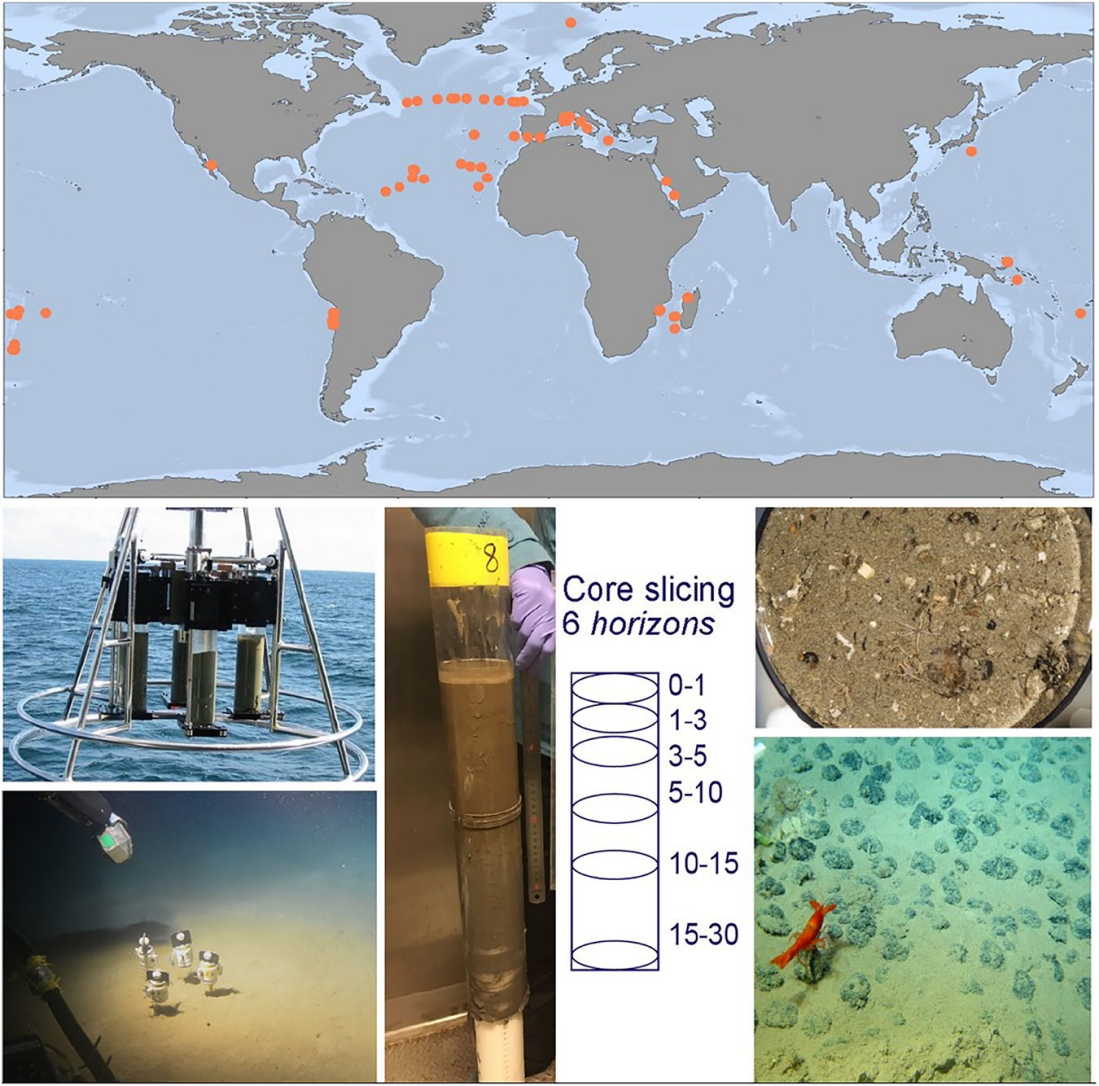
Sampling gear and sites. Maps of sediment samples collected for the Pourquoi Pas les Abysses? and eDNAbyss projects (see Annex 2 for a list of cruises). Pictures of the multicore (upper left, N/O Pourquoi Pas? © Ifremer) and a tube core sampling system deployed from a submersible (© Nautile, Ifremer), as well as from the slicing of cores and surface of a core sampled with multicore (© S. Arnaud-Haond) and a nodule area in the Clarion Clipperton zone (© Ifremer).

Like those earlier initiatives, obtaining a global appraisal of the diversity of life in the deep ocean requires a panoceanic project based on standardized sampling and analytical methodologies, which are applied both to the microbial realm and multicellular eukaryotes. This observation has been the main incentive of the ‘*Pourquoi Pas les Abysses?*’ project, initiated by Ifremer in 2016, to establish standardized pipelines from sampling to molecular and bioinformatic strategies. This project focused on the testing and validation of rigorous and standardized protocols to design a complete analytical pipeline from a sampling strategy^32^ to molecular^33,34^ and bioinformatic^35^ analyses of deep-sea diversity from the seafloor compared with the water column^36^, paving the way for integrative global-scale inventories. These protocols have been applied, as a proof of concept, to more than 1500 deep-sea samples collected worldwide at different depths (Figs. 1, 2) during the *eDNAbyss* project.

Launched in 2018, *eDNA was* integrated with the Tara Ocean to improve the methodological and biological coupling between studies of life in the deep seafloor and the water column^36^.

As a result, the consortium produced a large set of ‘omics’ data, including metagenomics, metabarcoding and capture by hybridization, and environmental data (sediment granulometry, organic matter, etc.). In this manuscript, we propose a complete overview of this dataset, the protocols used and developed to obtain it, which are listed in Tables 1–3 and detailed in the following sections, and the ways in which it can be accessed (Table 4)^37^.

**Table 1:** List of sampling gear used depending on the substrate targeted.

Substrate	Device used or *developed*	Approx. Volume collected	Picture
**Seafloor**			
Sediment	*Multicore	Per core Diameter 10 cm Height 30 cm 2,36 L	
	Push cores	Per core Diameter 6 cm Height 30 cm 0,85 L	
Hard substratum	*ELFES* scrapper	Volume of the sampling cell 5,5 L	
**Seawater**			
	*Cores supernatant	1 to 2 L	
Large volumes	*SALSA pump	Up to 35 000 L typically divided into 4–6 successive samples of 5000–8000 L	

*indicates those that do not require the availability of a submarine or ROV, i.e., that can be deployed directly from the boat.

**Table 2:** Target regions selected for metabarcodes with their associated taxonomic targets, with details of the expected fragment length, primer sequences, PCR conditions (volume, cycles, polymerase used with the manufacturer) and their original reference; all were submitted to a 2 × 250 bp sequencing length, except for 18S-V9, which required only 2 × 150 bp.

Locus	Target	Primer F	Sequence	Exp. size	PCR volume	PCR cycling	Taq Pol. & dNTP	Final Primer Conc.	Ref.
		Primer R							
COI	Eukaryote	mlCOIintF	GGWACWGGWTGAACWGTWTAYCCYCC	313	20	95 °C 10 min; 16 × (95 °C 10 s, 62 → 46 °C 30 s, 68 °C 60 s); 24 × (95 °C 10 s, 46 °C 30 s, 68 °C 60 s); 68 °C 7 min	1x Advantage 2 (Takara) + 0.175 mM final concentration of dNTPs Mix	0.5	^74^
	*(pref. metazoans)*	jgHCO2198	TAIACYTCIGGRTGICCRAARAAYCA						
18S V1-V2	Eukaryote	SSUF04	GCTTGTCTCAAAGATTAAGCC	450	25	98 °C 10 s; 40 × (45 °C 30 s, 72 °C 30 s); 72 °C 10 min	1X Phusion (Thermo)	0.4	^27^
	(*pref. metazoans*)	SSURmod	CCTGCTGCCTTCCTTRGA						
18S V4	Eukaryote	V4F (TAReukFWD1)	CCAGCASCYGCGGTAATTCC	380	25	98 °C 30 s; 10 × (98 °C 10 s, 53 °C 30 s, 72 °C 30 s); 17 × (98 °C 10 s, 48 °C 30 s, 72 °C 30 s); 72 °C 10 min	1x Phusion GC (Thermo)	0.4	^75^
	(*all*)	V4R (TAReukREV3)	ACTTTCGTTCTTGATYRA						
18S V9	Eukaryote	1389 F	TTGTACACACCGCCC	87–186	25	98 °C 30 s; 27 × (98 °C 10 s, 57 °C 30 s, 72 °C 30 s); 72 °C 10 min	1x Phusion GC (Thermo)	0.4	^76^
	(*all*)	1510 R	CCTTCYGCAGGTTCACCTAC						
16S V4-V5	Prokaryotes	515 f	GTGYCAGCMGCCGCGGTAA	411	20	98 °C 10 s; 29 × (98 °C 10 s, 53 °C 30 s, 72 °C 30 s); 72 °C 10 min	1X Phusion GC (Thermo)	0.4	^77^
	(*all*)	926r	CCGYCAATTYMTTTRAGTTT						
16S V4-V5	Prokaryotes	517 F	GCCTAAAGCATCCGTAGC	441	25	95 °C 3 min; 27 × (95 °C 30 s, 57 °C 45 s, 68 °C 60 s); 68 °C 2 min	1x Advantage 2 (Takara) + 0.175 mM final concentration of dNTPs Mix	0.5	^78^
	(*archae*)		GCCTAAAGCATCCGTAGC						
			GCCTAAARCGTYCGTAGC						
			GTCTAAAGGGTCYGTAGC						
			GCTTAAAGNGTYCGTAGC						
		958r	CCGGCGTTGANTCCAATT

**Table 3:** Set of probes targeting the 16S & 18S rRNA and COI genes used for CBH.

Name	Sequence 3′−5′
16S_1	CCAGACTCCTACGGGAGGCAGCAGTGGGGAA
16S_2	AAACTCCTACGGGAGGCAGCAGTGGGGAATCT
16S_3	CRAACSGGATTAGATACCCSGGTAGTCC
16S_4	AACAGGATTAGATACCCTGGTAGTCCACGCC
16S_5	GGGAGCAAACAGGATTAGATACCCTGGTAGT
16S_6	AACAGGATTAGATACCYTGGYAGTCCACGC
16S_7	AACAGGATWAGATACCCKGGYAGTCCAYRC
16S_8	ACTCAAAGGAATTGACGGGGGCCCGCACAAG
16S_9	CACAAGCGGTGGAGCATGTGGTTTAATTCGA
16S_10	CGCAAGDRTGAAACTTAAAGGAATTGGCGGGGGAGCAC
16S_11	GTTGGGTTAAGTCCCGCAACGAGCGCAACCC
16S_12	GAGAGGWGGTGCATGGCCGYCGYCAGYTCGT
16S_13	CATGGTTGTCGTCAGCTCGTGTCGTGAGATG
16S_14	TGTCGTCAGCTCGTGTCGTGAGATGTTGGGTTAAGTCCCGCAACGAGCSS
16S_15	TCGTCAGCTCGTGTYGTGAGRTGTTSGGTTAAGTCC
18S_1	AGGGCAAGTCTGGTGCCAGCAGCCGCGGTAA
18S_2	TCTGGTGCCAGCAGCCGCGGTAATTCCAGCT
18S_3	TGCCAGCAGCCGCGGTAATTCCAGCTCCAAT
18S_4	CGCGGTAATTCCAGCTCCAATAGCGTATATT
18S_5	GAGGGCAAGTCTGGTGCCAGCAGCCGCGGTAATTCCAGCTCCAATAGCGT
18S_6	GTCCCTGCCCTTTGTACACACCGCCCGTCGC
18S_7	GATTACGTCCCTGCCCTTTGTACACACCGCC
18S_8	TTGATTACGTCCCTGCCCTTTGTACACACCGCCCGTCGCTA
18S_9	GAGCCTGCGGCTTAATTTGACTCAACACGGG
18S_10	AAGGAATTGACGGAAGGGCACCACCAGGAGT
18S_11	GGAAGGGCACCACCAGGAGTGGAGCCTCGGCTTAATTTGACTCAACACGG
18S_12	TGGTGGTGCATGGCCGTTCTTAGTTGGTGGA
18S_13	TGGGTGGTGGTGCATGGCCGTTCTTAGTTGGTGGAGTGATTTGTCT
18S_14	GCAATAACAGGTCTGTGATGCCCTTAGATGT
18S_15	AAACTTAAAGGAATTGACGGAAGGGCACCAC
18S_16	GGGGGAGTATGGTCGCAAGGCTGAAACTTAA
18S_17	GTATGGTCGCAAGGCTGAAACTTAAAGGAATTGACGGAAGGGCACCACCA
COI_1	ATYGTHACDGCMCAYGCHTTYRTTATAATC
COI_2	TTYCCHCGDATRAATAAYWTAAGWTTTTGR
COI_3	AACDGAYCGAAATYTAAATACHDCHTTYTT
COI_4	TAYCAACAYYTVTTYTGATTYTTYGGNCAT
COI_5	TAGGHTTTRTHGTRTGRGCHCAYCAYATAT
COI_6	GCHACWATAATTATTGCWGTHCCNACVGGR
COI_7	TTCYTATTYACARTDGGDGGVYTAACWGGA
COI_8	TCAYGAYACHTACTATGTDGTHGCHCAYTT
COI_9	TTATGTNGTDGCHCAYTTYCATTATGTDYT
COI_10	ATGTNGTWGCNCATTTTCAYTATGTDCTHT
COI_11	CTCAYCATATRTTYACNGTWGGDATRGAYG
COI_12	CCCWGAYATRGCHTTCCCHCGWATRAAHAA
COI_13	TTCHTCWATTYTAGGDGCHATYAACTTYAT

**Table 4:** Organization of the eDNAbyss repository at ENA, with the accession number for the umbrella project eDNAbyss and the details of the accession numbers for each nested bioproject.

Project type	Umbrella project	Nested bioprojects					
	eDNAbyss project	Metabarcoding of eDNAbyss sediment samples	Metabarcoding of eDNAbyss water samples	eDNAbyss Metagenomics on sediment samples	eDNAbyss Metagenomics on water samples	Ammonia-oxidizing archaea of South Pacific abyssal and hadal surface sediments	Capture by hybridization in the deep -sea
Accession number	PRJEB39225	PRJEB33873	PRJEB37673	PRJEB57745	PRJEB57853	PRJEB60556	PRJNA693247
Metabarcoding							
Prokaryotic 16S		814/1,401	39/143				
Archae16S		278/855	−/104				
18SV4		121/477	33/102				
18SV9		98/805	−/129				
18SV1V2		193/1,208	22/153				
COI		174/582	22/101				
Metagenomics				205 (60)/205	43/43		20/40
MAGs						47/47	

All accession numbers are links that can be clicked to access the corresponding ENA record. The numbers indicate the assays released as of May 2025 relative to the total number of samples analyzed. Assays associated with previous publications for technical development or microbiology, when different from the numbers released, are shown in brackets.

As a result, we hope that this manuscript can be adopted by other teams to extend the FAIR (Findable, Accessible, Interoperable and Reusable) dataset and workflow developed from sampling to bioinformatics data analysis to gather, step by step, data collection representative of deep-sea biodiversity at global taxonomic and geographical scales.

## Methods

### Sampling and selection of environmental samples

#### Substrate

Compared with larger core sampling systems, tube-core sampling systems can provide replicates of intact, stratified and standard volumes of sediment samples from a single deployment that are easy to slice (e.g., compared with box-core samples, which tend to lead to the loss or mixing of the precious first centimetres where most meiobenthic taxa are usually encountered, Bett *et al*.^38^). These core systems are also fairly easy to disinfect between each deployment, limiting the risk of contamination between sites. Using tube cores as sampling units also offers researchers the flexibility to sample either using multicore samplers deployed directly from research vessels or to obtain more precisely positioned cores deployed from a submersible (Fig. 2).

As both prokaryotes and eukaryotes present a heterogeneous distribution with depth in the sediment, a fine-grained understanding of their depth segregation can be obtained by sampling vertically, i.e., slicing cores in different ‘*horizons’*. Vertical slicing is important for characterizing not only the distribution of living communities but also the interplay between living compartments, particularly prokaryotes, and biogeochemical processes. First, most organic matter and biodiversity are usually found in the first layers of sediments: homogeneizing distinct layers leads to a loss of information but also to the dilution and consequent undercharacterization of living organisms in the biomass-poor layers. Second, accounting for biogeochemical stratification is ideal in specific cases, such as the characterization of bacterial communities and their functions^39,40^. However, such fine-grained *in situ* information is seldom available. In addition, it is not expected to be related to the distinct kingdom and taxa that form deep-sea benthic communities: macrofauna may dig across layers of sediment, whereas the depth range of microbial and meiofaunal taxa may be more constrained by the sedimentation rate. It is therefore important to acknowledge that no ‘*jack of all trades*’ exists in terms of core slicing. The choice of the number of slices and depth of each horizon is a trade-off between community characterization across the Tree of Life and the resources available for each site. While adopting a similar strategy is essential to reach interoperability of data, different slicing strategies might be considered in some particular cases, depending on the sedimentation rate and taxa targeted. These methods may allow one to unravel fine-grained patterns and, second, to avoid blurring contemporary community assessments with ancient sedimentary DNA archived in the sediment (i.e., *seda*DNA^41^). For example, in some areas where the sedimentation rate is very low, ancient deposits may be found only a few cm downcore and thus mostly deliver *seda*DNA. This was exemplified during a study of multicore samples from the South Atlantic, where sediments 30 cm below the sediment–water interface were aged between 11,000 and 30,000 years^42^.

Two main paths can be considered for standardizing slicing: accounting for biogeochemical data when available or arbitrarily defining a standard scheme inspired by the literature. We applied the former strategy, driven by the biogeochemical characteristics already gathered (before sampling) in hadal areas characterized by very high sedimentation rates, to match the environmental gradient and biotic characterization of prokaryotes^39,40^. In fact, the number of living organisms present at increasing sediment depths is strongly dependent on sedimentation rates, which are highly variable among trenches but are, on average, greater than those in surrounding deep-sea areas^43^. Such fine cm slicing allows finer grain characterization of the living and ancient communities and can be reconciled with coarser-grained slicing by pooling sediment slices after having conditioned finer-grained subsamples, as was done in *eDNAbyss* for samples from hadal trenches^44,45^.

In the most common case where no biogeochemical data are available a priori and/or a broad range of taxa is targeted, a diversity of vertical sampling schemes has thus been adopted over the years, with some common trends in the abyssal areas. First, living prokaryote communities can be found even deeper than 30 cm, while for animalia, the focus is usually on the first 10 cm, where most biomass and diversity are encountered. Second, in line with the observation of up to 90–95% animalia fauna in the first 3–4 cm in the abyssal areas of the North Atlantic^46,47^, a decreasing resolution is usually adopted with depth in the sediment. For example, in the Pacific Clarion Clipperton nodules area, the International Seabed Authority recommended these observations (with a slicing of the first 10 cm by 0–2, 2–5, and 5–10 cm) to standardize approaches adopted by different contractors, despite some research teams adopting slight variations to ensure temporal comparability of samples with more ancient samples (0–3, 3–5, and 5–10 cm horizons^48^). We sampled cores downwards to the maximum depth obtained to accommodate both prokaryote and eukaryote sampling with a slightly higher slicing resolution to offer a better trade-off among the diversity of sites explored. As a result, the most common strategy for sediment sampling during *eDNAbyss* was to deploy triplicates of standard 10 cm diameter cores that were 30–50 cm long and separated by several tens of centimetres and were cut out in vertical increments (all depths relative to the core top): 0–1, 1–3, 3–5, 5–10, 10–15 and 15–30 cm following other studies in abyssal^49–51^ and hadal^52^ areas and general recommendations to sample in the deep sea^53^.

In terms of freezing and long-term storage, the best way to condition samples was to flatten each fraction after homogenization in a Ziplock bag. This allows faster freezing, optimal space occupation in ultrafreezers and transport boxes, and above all, easy subsampling for analysis and extraction without thawing the whole sample (just breaking a corner piece equivalent to the weight needed).

**Fig. 3:**
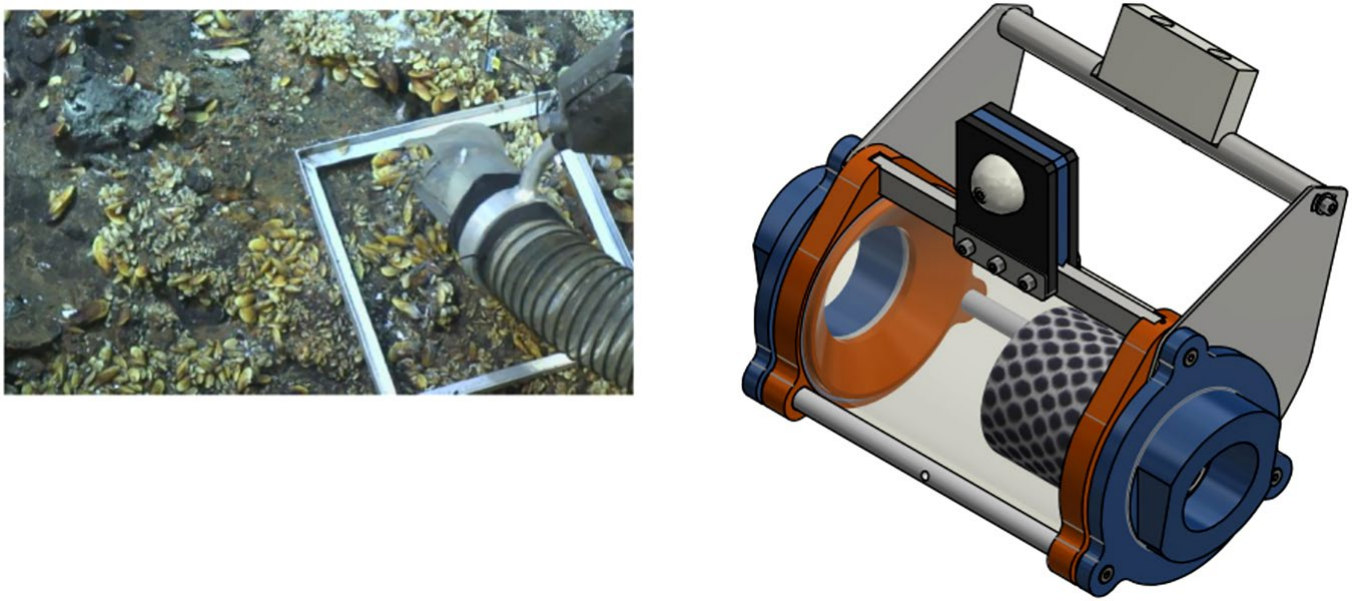
ELFES is a dedicated hard substratum sampler. Left: the nozzle of the sampler that scrapes the fauna on hard substratum, here on an active hydrothermal sulfide structure during the Momarsat 2017 cruise (10.17600/17000500). Right: drawing of the cartridge that directly collects the fauna.

With respect to the standardized protocol proposed for sediment samples, importantly, the *“Pourquoi Pas les Abysses?”* project also developed new technologies to facilitate quantitative sampling from hard substrata. In the absence of a dedicated tool to quantitatively sample faunal communities associated with hard substrata^23^, the deep-sea laboratory at Ifremer developed the ELFES (“*EchantiLlonneur de Faune de Substrat dur*”) sampler prototype (Fig. 3)^54^. This system includes a rake to scrape the substratum combined with a dedicated powerful suction device to collect all available benthic fauna quantitatively in a dedicated cartridge equipped with 55 µm mesh. The ELFES will be integrated into future versions of French submersibles. Metabarcoding analyses of these samples revealed good performance in detecting taxa associated with vent communities on hydrothermal and inactive structures^55^.

#### Water

Owing to the oligotrophic nature of many deep-sea water masses and the high heterogeneity of biomass distribution in abyss, many uncertainties exist as to the minimal amount of DNA one may be able to obtain depending on the volume of sediments, rocks or water processed. Here, we targeted whole communities, including metazoans and microorganisms. We hypothesized that sampling water could mitigate the heterogeneous nature of the abyssal landscape if the benthic community at the deep-ocean floor (DOF) surface left a signature in the surrounding water layers^32^.

Three main paths were followed to sample aboveground water and investigate its ability to deliver hints on pelagic but also benthic biodiversity through the use of large versus small volumes of water, with independent gear (Niskin or hermetic sampling boxes) versus cores (using the supernatant) in the case of smaller volumes, all collected at a maximum of 1 m above the seafloor.

**Fig. 4:**
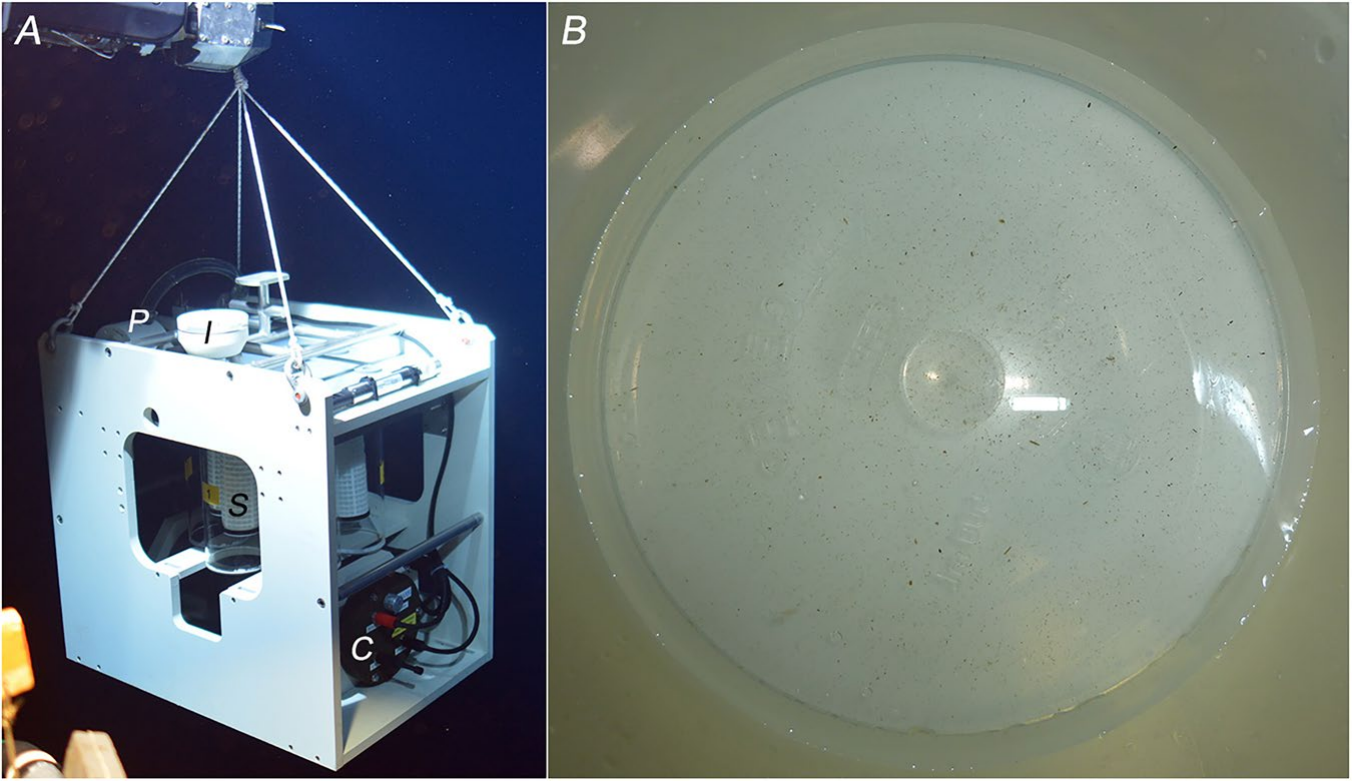
SALSA sampling. A high-volume SALSA pump (developed at Ifremer and based on McLane) was deployed on the seafloor by the HOV Nautile at a depth of 3500 m along the Mid-Atlantic Ridge (BICOSE 2 cruise 10.17600/18000004, AMIGO cruise series 10.18142/247). I: water intake, P: pump, S: sampling bowl, C: controller housing containing batteries and a controller board. **b**) Typical amount of material collected in a 5000 L sample collected from the bottom of the Mediterranean abyssal plain (ESSNAUT 2016 cruise, 10.17600/16000500, AMIGO cruise series 10.18142/247). The material was concentrated from a sampling bowl on a 15 cm diameter dish.

First, large volumes (thousands of L) were sampled during tests at the beginning of the project via the recently developed “Serial Autonomous Larval Sampler (SALSA)”, a McLane WTS-LV sampler customized at Ifremer, Brest, France, to allow replicate samples of large volumes of water to be prefiltered *in situ* (Fig. 4). The pump itself is placed downstream of a set of six 2.8 L sampling bowls fixed on a rosette holder underneath a rotator plate. This plate sequentially aligns each bowl with the water intake, and prefiltration occurs through the outlet of each bowl to retain only particles >20 µm. This system provides *in situ* concentrations of particles across large volumes of water (up to 35 000 L in one deployment, split among sampling bowls according to a user-defined program). The SALSA pump was initially modified to target particles >20 µm and now allows filtration of >5 µm particles, delivering samples dedicated to metazoan inventories. The SALSA can be deployed directly on the bottom, with its water intake located approximately 80 cm above the seafloor. The pump is usually positioned with a submersible, but deployment from the research vessel is also possible via a cable. In this case, an acoustic beacon is used to obtain the pump position, and sampling can be within 10 m from the seafloor at best. The prefiltered water from the SALSA was refiltered on board a polycarbonate filter membrane with a 2-μm mesh size (Millipore, Burlington, MA, USA; ref. TTTP04700).

For the second and third options focusing on smaller volumes, collection was performed via 7.5 L sterile and isotherm sampling boxes^56^ filled on the bottom by the submarine Nautile (EssNaut cruise), and above-ground water was collected from the sediment cores (supernatant, usually 1–2 L). Small volumes were initially filtered onboard using a peristaltic pump system and a succession of 47 mm polycarbonate filters with 20 μm, 2 μm, and 0.22 μm pore sizes (Millipore, Burlington, MA, USA, with the respective references NY2004700, TTTP04700, and GTTP04700). Owing to the oligotrophic nature of most deep-sea water layers, the filters were likely not saturated. Filtering of small volumes was thus eventually performed directly on polycarbonate filters of 47 mm and 0.22 µm, allowing the targeting of both metazoans and microbial organisms from the same samples (Table 2).

Both sampling methods can be considered complementary, with large volumes of several hundred to thousand litres prefiltered on coarser sizes (20 or 5 μm mesh size) collected with SALSA favouring metazoans and smaller volumes of 2 to 7.5 L (20 down to 0.2 μm) collected with the sampling box or using the core supernatant and filtered on smaller mesh sizes, allowing better characterization of prokaryotes. The comparison of communities in water samples from small volumes collected in boxes or large volumes collected with SALSA and those from sediment samples clearly revealed that independent water samples, regardless of volume and distance from the DOF, revealed only pelagic communities and failed to characterize benthic life^32^. As a result, the dataset includes benthic communities in the DOF characterized on the basis of sediment sampling at all sites. Data from the supernatant were also gathered from some of the cruises, as the water on top of sediment cores can deliver results comparable to those found in the first centimetre of sediment cores (pers. obs.), with an additional value to the characterization of superficial benthic communities and sinking pelagic communities.

#### Association of metadata

##### Basic metadata

For each sampling event, basic metadata were recorded from the oceanographic vessel, including GPS coordinates, depth, date, name of the cruise (with doi as the cruise report), name of the ship, device used for sampling, marine region code from the world marine regions (https://www.marineregions.org) and biome and environmental features classified from the environmental ontology database (ENVO, https://bioportal.bioontology.org/ontologies/ENVO?p=classes&conceptid=ENVO%3A00000428).

The distance to the nearest coastline for each site was determined via the *sp*, *rgeos*, *maptool*, *rgdal* and *raster* packages in R, together with coastal distribution data from naturalearthdata.com.

##### Sediment characterization

At each site, all horizon samples were analysed for granulometry (one core per site), water content and organic matter content (two cores per site).

First, to measure the humidity, approximately 2 g of material was introduced into a clean container before being placed overnight in an oven at 100 °C. The containers were weighed again at the exit of the oven, placed in an oven for 4 hours at approximately 550 °C, and weighed again. The humidity and loss on ignition at 550 °C were then calculated, and the water (W) and organic matter (MO) contents in each sample were estimated.

Second, granulometry, by laser diffraction through wet dispersion, was used to determine the distribution of particle size across many size classes, from 0.02 to 2,000 µm, via a laser particle size analyser MASTERSIZER 3000 from MALVERN Instruments. The particle size measurements were carried out in flowing water at 2000 rpm. For each sample, a spatula tip was added to the water to break possible agglomerates of material, and ultrasonication was applied for 30 s at 100% power. After 30 s of stirring without ultrasonication to stabilize, a minimum of 4 measurements were made per sample. For each site, one to two cores from each horizon were analysed via laser granulometry, and at least two cores were analysed for water and organic matter contents. The following metrics were extracted:

D[4:3] is a metric that considers the volume mean diameter and is based on the frequency distribution of particle sizes:

1 $$D[4,3]=\frac{\mathop{\sum }\limits_{1}^{n}{D}_{i}^{4}{v}_{i}}{\mathop{\sum }\limits_{1}^{n}{D}_{i}^{3\,}{v}_{i}}$$

where *D*_i_ is the geometric mean for size class *i* (the square root of Dmax X Dmin of this particular size class) and v_i_ is the percentage of this size class in the sample. This is considered the ‘volume mean diameter’ and will be designed as the mean particle size or *D*_43_ in the text.

Dspan is a metric that describes the width of the sediment size distribution as follows:

2 $${D}_{{span}}=\frac{{{\rm{D}}}_{v0.9}-{{\rm{D}}}_{v0.1}}{{{\rm{D}}}_{v0.5}}$$

With Dv_0.1_, Dv_0.5_ and Dv_0.9,_ the maximum sizes of the particles are less than 10, 50 and 90% of the distribution, respectively.

To describe the sediment properties for each sediment layer at each site accounting for all replicate measurements, a label was assigned to each sample, designating the combination of site and horizon to which it belonged. The mean and standard deviation of each parameter were estimated for each site and horizon. Calculations were performed via the R package *dplyr* with the functions *group_by* and *mutate_each*.

##### Data from global databases

We used databases to gather metadata associated with the samples collected. All the data are listed in Supplementary File 1 Table S1.

The sediment type category data (25 categories in 8 broad categories, see Annex I) were obtained from the French Naval Hydrographic and Oceanographic Service (SHOM, https://data.shom.fr/) 2011 World Sedimentary Chart v7.1, which consists of 35 shapefiles covering the world oceans. The data contained in these files were aggregated to the sampled sites (Supplementary File 2).

The Bio-Oracle database (https://www.bio-oracle.org/) of oceanographic data measured *in situ* or derived from satellite observations and modelling) was used to obtain ten benthic environment variables and one surface water variable (Supplementary File 1, Table S1). The versions of the database used were the Bio-Oracle database v2.0 (for salinity only) and v2.1^57,58^, with monthly averages for the period 2000–2014 on *rasters* and a common spatial resolution of 0.08°. Most of the variables chosen were hypothesized to influence the distribution of deep-ocean communities (e.g., temperature); however, we also extracted other variables without any a priori knowledge of their potential influence on communities (e.g., nitrate).

For each environmental variable, the mean value and standard deviation at each site were calculated from the parameter values available for the benthic layers of the ocean for the years 2000–2014. Owing to the relatively coarse resolution (0.08°), a given cell may be associated with heterogeneous depth values, and the mean and standard deviation of each parameter were thus calculated from the maximum, minimum and mean values of the parameters at the maximum, minimum and mean depths of each sampled area. Nine values were therefore eventually used to calculate the mean and standard deviation, which is the most robust possible calculation given that complete distributions of parameter values are not available on Bio-Oracle.

For surface primary productivity, we calculated the mean and standard deviation from the mean, minimum and maximum values of surface primary production. The mean and standard deviation values for each of these variables were then aggregated to the sampled sites (*extract()* function in the raster package).

Data were first used to evaluate the relationship between the success/failure of library construction and sequencing and environmental data. They will later be used to screen for the possible existence of a relationship between the identity of some taxa and the community composition with the available environmental descriptors of the habitat.

### Handling of samples

The environmental samples were kept at −80 °C onboard, in Ziplock bags for sediments, and in Falcon bags, Ziplock bags, sterile cryotubes or Petri dishes for water filtration. They were dispatched to the laboratories on dry ice and kept at −80 °C until DNA extraction or sediment analysis (for granulometry, organic matter, etc.).

All ziplock bags or other containers contained a unique identifier indicating the cruise-site-core/filter-horizon/mesh_size, associated with a database containing all associated metadata (date, GPS coordinates, depth, etc.) and posted on the European Nucleotide Archive (see the Data Records section).

### Nucleic acid extraction and quality control of nucleic acids before the production of libraries

#### Sediment: The choice of DNA over RNA

While an exponential decay of eDNA has often been observed in seawater^59–62^, long-term archiving of DNA has been reported in sediments^63–65^. Several hypotheses have been proposed in the literature to determine which nucleic acids (RNA or DNA) more faithfully reflect contemporary communities. The issues associated with possible DNA archiving strongly depend on the size of the DNA fragments targeted and the age of the DNA (DNA that has been archived for longer periods is expected to be more fragmented)^42,64,66,67^. In the abyssal sediment of the deep Atlantic, the size of successfully amplified fragments decreased from 1000 bp at the sediment surface to 250 bp at 10 cm and less than 100 bp at lower horizons^42^, and studies in sediments from Australia and Antarctica reported that ancient DNA fragment sizes are usually smaller than 100 to 70 bp^64,68^. To target present rather than past members of the communities, we tested various DNA and RNA extraction methods. We compared protocols favouring intracellular DNA^33^ and those selecting longer fragments over shorter fragments (i.e., bead selection to select fragments > 1000 bp or EtOH precipitation of raw DNA extracts to favour longer fragments). In line with previous results focused on prokaryotes^67^, our results showed that filtering DNA extracts to retain only DNA fragments larger than 1,000 bp did not influence biodiversity inventories^33^. Therefore, the metabarcode fragments sized, in this experiment, 313–450 bp were amplified from fragments longer than 1000 bp. The effects of the extraction kits were comparable, or even more important for metazoans, to the nucleic acid (DNA *vs*. RNA) effect, which was likely due to the amount of material extracted (2 g for RNA *vs*. 10 g for DNA), and larger samples of sediment apparently led to a better representation of biodiversity. Given the logistical difficulties associated with the preservation and manipulation of samples when working with RNA and the fact that studies have also reported long-term archiving for RNA^69,70^, the decision was made to focus on DNA^33^. Finally, the use of ~10 g of homogenized sediment with the PowerMax Soil DNA Isolation Kit (MOBIO Laboratories Inc.; Qiagen, Hilden, Germany) was selected for large-scale data production, as larger sediment volumes were more adequate for obtaining a comprehensive census of life, particularly for eukaryotes.

A frozen piece (~10 g) of sediment was taken from the original ziplock and transferred to a 50 ml Falcon tube. Once the sediment had thawed, all the extraction steps followed the manufacturer’s manual, except for the last step. During elution, the buffer was left for 10 min on the filter at room temperature instead of being immediately centrifuged to increase the DNA yield.

The sampling/field controls consisted of empty bags preserved during the cruises. They were processed by injecting Milli-Q water or the first buffer of the kit in a plastic bag before transferring it into the first 50 ml falcon tube of the extraction kit. The laboratory extraction controls were performed by adding Milli-Q water to the first tube of the extraction kit.

#### Hard substratum

Few trials were performed on hard substratum samples for which a particular protocol was developed. These data can be found in Cowart *et al*.^55^ For sample recovery onboard the ship and before fauna were sorted, approximately 9–10 g of substratum, debris and pebbles were collected directly for each quadrat collection from the sampling boxes or ELFES cartridges via sterilized (using a 10% bleach solution) and rinsed (with Milli-Q water) tweezers and spoons and then placed in sterile 50 ml centrifuge tubes. These samples were stored at −80 °C until eDNA extraction. Genomic DNA was extracted from approximately 10 g of substratum via the Qiagen PowerMax R© Soil DNA Isolation Kit in accordance with the manufacturer’s instructions. The eDNA extractions were performed under a Captair R© bio BioCap DNA/RNA workstation (Rlab, Rowley, MA, United States) with UV sterilization and filtered airflow. Tools and equipment were UV sterilized and washed with a 50% bleach solution following protocols from Goldberg *et al*.^71^ The samples were handled via dedicated eDNA pipettes fitted with filter tips. Negative (extraction starting with an empty tube) and positive extraction controls (using local shallow water sand from the “Plage du Dellec”, Plouzané, France)) were included in each extraction series. The concentration of each sample was assessed via a Qubit R© dsDNA HS Assay Kit (Thermo Fisher Scientific, Waltham, MA, United States). The samples were sent to Mr DNA Lab (Shallowater, TX, United States) for amplicon generation, library preparation and subsequent metabarcode sequencing. The positive controls contained measurable amounts of DNA; none of the negative controls showed any sign of contamination.

#### Sample biobanking

Once extracted, the DNA was split into one “working” aliquot (2 mL) and two “biobanking” aliquots (1.5 ml). The “working” aliquot was sent to Genoscope (French National Center of Sequencing, Evry, France) for immediate and future analyses, whereas the two “biobanking” aliquots were frozen (−80 °C) in cryotubes for preservation at the Ifremer-Marbec laboratory in Sète. The remaining sediments were also preserved in Sète. The “working” aliquot and the two “biobanking” aliquots share the same sample accession number and therefore the same metadata and environmental context provided by BioSamples.

This archive, along with the metadata database with unique IDs detailed below, allows for their future use for other purposes and may be accessed by contacting the corresponding authors.

#### Water filters

All nucleic acid extractions were carried out by the Genoscope Laboratory (Evry, France) via the same protocol as that described by Alberti *et al*.^72^ for the Tara Oceans water samples and in Brandt *et al*.^32^ The protocol was set up for the joint extraction of DNA and RNA, with a first step of cryogenic grinding of membrane filters followed by nucleic acid extraction via NucleoSpin RNA kits combined with the NucleoSpin RNA/DNA buffer set (Macherey-Nagel, Düren, Germany). Briefly, the extraction consisted of the following steps:

Grinding: Each polycarbonate filter was placed in a grinding vial with 1–2 ml of lysis buffer (Macherey-Nagel RA1 lysis buffer with 1% β-mercaptoethanol—Sigma, St. Louis, MO-) and ground with a Freezer/Mill instrument (6770 or 6870 Freezer/Mill; SPEX SamplePrep, Metuchen, NJ) via the following program: 2 min of precooling, one grinding cycle with 10 pulses per second for 1 min followed by 1 min of cooling, and a final grinding cycle at 10 pulses per second for 1 min.

Lysis: Cryoground powder was then transferred to a large capacity NucleoSpin filter (Nucleospin Filters XL, ref 740605), and the remainder was recovered from the grinding vials by adding 1 ml of lysis buffer to the NucleoSpin filters. The solution was left on the filter for ~30 min to ensure gravity flow. The eluate was transferred to a 15 mL Falcon tube before centrifugation at 1,550 × g (20 min at 20 °C). After the addition of 500 μL of lysis buffer to the eluate, a second centrifugation step was performed at 1,550 × g (3 min at 20 °C).

Precipitation and washing: One volume of 70% ethanol was then added, and the final mixture was placed onto a NucleoSpin RNA Mini spin column, where it was washed twice with 500 μL of DNA wash solution. The columns were dried for 3 min at room temperature.

Elution: DNA was eluted in three successive elutions with a total of 100 μL of DNA elution buffer and stored with 1 mM EDTA in sterile 1.5 mL LoBind Eppendorf tubes at −20 °C.

RNA isolation was subsequently performed on the same NucleoSpin RNA Mini spin column through the addition of 10 μl of rDNase to degrade any residual DNA, 90 μl of reaction buffer for rDNase, 15 min of incubation at room temperature, and two steps of washing with RAW2 and RA3 buffers. The RNA was then eluted in 60 μl of RNase-free water and stored in sterile 1.5 mL Ambion Non-Stick RNase-free tubes at −80 °C.

#### DNA Qualification and quantification

For water, sediment and hard substratum samples, DNA was quantified via a dsDNA-specific fluorometric quantitation method via a Qubit 2.0 fluorometer instrument, sequentially via the Qubit dsDNA BR (broad range) and HS (high sensitivity) assays (Thermo Fisher Scientific, Waltham, MA). The first assay was performed with dsDNA BR to roughly evaluate the concentration. To obtain more precise estimates, an aliquot of the DNA mixture was then diluted according to the BR assay values to bring the concentration within the concentration threshold range of the HS kit. Two HS assays were then carried out using this diluted DNA solution, and the concentration was estimated as the average of the concentrations from these 2 assays.

The RNA was quantified via RNA-specific fluorometric quantification on a Qubit 2.0 fluorometer via a Qubit RNA HS assay kit. The concentration was calculated by averaging the concentrations from 2 assays. The RNA quality was evaluated via capillary electrophoresis on an Agilent Bioanalyzer via an RNA 6,000 Pico LabChip Kit (Agilent Technologies, Santa Clara, CA).

### Metagenomic library construction and sequencing

#### Library construction

Depending on the amount of DNA available, 1.7 to 12.5 ng of genomic DNA was sonicated to obtain fragments of approximately 350 bp via a Covaris E220 instrument (Covaris, Woburn, MA, USA). For more than 82% of the samples, the input was 12.5 ng. For the remaining 18%, which presented a concentration that was too low, the input varied from 1.7 to 11.9 ng. Fragments were end-repaired by the 5′ → 3′ polymerase and 3′ → 5′ exonuclease activities of T4 DNA Polymerase to obtain blunt-end DNA, 3′-adenylated to add a nonmodelled nucleotide to the 3′ end of a blunt nucleotide, and NEXTflex PCR-free barcode adapters (Bioo Scientific, Austin, TX, USA) were added via the NEBNext® Ultra II DNA Library Prep Kit for Illumina (New England Biolabs, Ipswich, MA, USA). Ligation products were purified with Ampure XP 0.8 Vol (Beckmann Coulter, Brea, CA, USA). For some samples based on the bioanalyzer profile, a second purification by Ampure XP 1Vol was necessary to remove all adapters. The resulting DNA fragments (>200 bp) were subsequently amplified via PCR (2 PCRs, 14 cycles) via Illumina adapter-specific primers and NEBNext® Ultra II Q5 Master Mix (NEB).

#### Quality control

All libraries were subjected to size profile analysis with an Agilent 2100 Bioanalyzer (Agilent Technologies, Santa Clara, CA, USA) to determine the absence of an adaptor peak and the concentration of DNA. If the library profile showed no adaptor peak and the concentration was >5 ng/μl, the library was considered valid. If the library profile had a peak of adaptors and the concentration was >10 ng/μl, we performed purification with AMPure bids at 1 volume to eliminate the adaptors.

When the library profile did not show an adaptor peak and the concentration was approximately 5 ng/μl, we estimated the concentration in nmol/L as a function of the fragment size. If the results were ≥10 nM, the library was considered valid; otherwise, it was discarded.

The validated libraries were then subjected to qPCR quantification via the KAPA Library Quantification Kit for Illumina Libraries (KapaBiosystems, Wilmington, MA, USA). These quantities were used to prepare the stock solutions for the flowcell preparation.

#### Illumina sequencing

Quality-control-validated water and sediment metagenomic libraries were sequenced via 151-bp pairwise read chemistry on an Illumina NovaSeq 6000 sequencer via S4 Flowcells (Illumina, San Diego, CA, USA). On average, 145 million useful paired-end reads were obtained per sample.

### Metabarcode library construction and sequencing

Two protocols were used during the project. Before 2020, the samples were processed via a strategy referred to hereafter as no-BID, and from 2020 onwards, the samples were processed via a strategy referred to as BID. This change was introduced to reduce cost and handling times. Whereas the non-BID strategy uses primers whose sequences correspond exactly to the published primer sequences, the BID strategy uses primers composed of the published primer sequences plus a small sequence of 8 nucleotides at the 5′ end, corresponding to a barcode identifier (BID). We used 12 BIDs (the same as those associated with forwards and reverse primers) to allow reliable reassignment of sequences to samples after demultiplexing.

For the no-BID strategy, the next step after the first PCR step was to construct one library per PCR product, i.e., per sample, whereas the BID strategy allowed us to construct a library from 12 samples by first creating a pool of 12 PCR products.

These two strategies are described in detail in Figure S1 of the Supplementary Figures and Tables section in Belser *et al*.^73^.

The PCR amplification, library construction and sequencing protocols are described in detail in the following sections, and Table 2 summarizes the key information.

#### PCR Amplification and generation of gene amplicons

The MetaB strategy of the eDNAbyss project involved studying 3 major lineages of the tree of life—eukaryotes (metazoans—now Animalia—and protist organisms), archaea and bacteria—using 6 sets of primers to target 5 marker genes—COI^74^, 18SV1V2^27^, 18SV4^75^ and 18SV9^76^—to target eukaryotes and 16SV4V5—to target prokaryotes^77,78^. Each of these marker genes was amplified as described below and in Table 2 via 2 types of primers, with or without BIDs.

Amplicons targeting eukaryotic and prokaryotic barcode regions were generated via specific primer pairs, and the optimized PCR protocols are summarized in Table 2. Each PCR used ≤ 2.5 ng of total DNA and included 3% DMSO and 1X Polymerase Mix. To reduce intrasample variance and ensure sufficient yield for Illumina sequencing, triplicate (or quadruplicate for COI) PCRs were performed. The PCR products were purified via AMPure XP beads at the specified volumes (Beckmann Coulter Genomics: 1x for all markers but 18S-V9, for which 1.8x is necessary). Aliquots of the purified amplicons were quality checked via an Agilent Bioanalyzer (DNA High Sensitivity LabChip Kit) and quantified via a Qubit 2.0 fluorometer (Qubit RNA HS Assay Kit).

#### PCR product quality control

Aliquots of purified amplicons were run on an Agilent Bioanalyzer via the DNA High Sensitivity LabChip Kit to check their lengths and quantified with a Qubit 2.0 fluorometer via the Qubit RNA HS Assay Kit.

#### Library construction

For both BID libraries and non-BID libraries, the same protocol was applied. The only difference lies in the input material. The preparation of the no-BID and BID libraries was carried out using 100 ng (or less, when the concentration of the PCR products did not allow it) of purified PCR products and an equimolar pool of 8 to 12 purified PCR products, respectively. Most of the sequencing libraries were obtained via high-throughput automated instruments and, by hand, when the first trial, which involved the use of a robot, did not yield a valid library. One hundred nanograms (or fewer) of purified amplicons or pooled amplicons were directly end-repaired, A-tailed and ligated to Illumina adapters on a Biomek FX Laboratory Automation Workstation. The libraries were then amplified (2 PCRs, 12 cycles) via the Kapa Hifi HotStart NGS library amplification kit under the same cycling conditions as those used for the metagenomic libraries and cleaned via AMPure XP purification (1 volume).

#### Library quality control

The libraries were first quantified by Quant-it dsDNA HS using a Fluoroskan Ascent instrument (Thermo Scientific), followed by qPCR with the KAPA Library Quantification Kit for Illumina libraries (Kapa Biosystems) via an MXPro instrument (Agilent Technologies). Library profiles were evaluated via a high-throughput microfluidic capillary electrophoresis system (LabChip GX, Perkin Elmer, Waltham, MA). The samples were subjected to the same size profile analysis and quantification as described for the metagenome libraries.

#### Illumina sequencing

Metabarcode libraries are often characterized by low-diversity sequences at the beginning of the reads related to the presence of sequences of primers used to amplify tags. Such low-diversity portions can interfere with correct cluster identification, resulting in a drastic loss of data output. In order to minimize the impact on run quality, these libraries were used with lower concentrations (8–9 pM instead of 12–14 pM for standard libraries) and increased PhiX DNA spike-in (20% for MetaB instead of the classically used 1% for MetaG). Sequencing was performed on HiSeq 4000 or HiSeq 2500 for the No-BID strategy libraries and NovaSeq 6000 instruments for the BID strategy libraries (Illumina) in paired-end mode. Read lengths were chosen to meet the bioinformatics analysis requirements.

Illumina real-time analysis (RTA) software (Code availability 1) was used for primary analysis during the sequencing run to analyse images and cluster intensities and remove low-quality data (i.e., reads below the thresholds imposed by the Illumina chastity filter). Furthermore, it performs base calling and provides a Phred quality score (Q score) indicating the probability that a given base is called incorrectly. The bcl2fastq Conversion Software v2.20.0.422 (Code availability 2) was used to convert the raw BCL files generated by RTA to fastq demultiplexed files allowing one mismatch when identifying the index sequence. For the metabarcoding data generated with the BID libraries, a second demultiplexing step was performed to assign the amplicon sequences to the correct samples and remove the 8-bp BID sequence. The sequence of each BID was searched in paired-end read files with Cutadapt v.1.18 (Code availability 3). An amplicon sequence was assigned to a given BID if it was found in both sequencing files (READ 1 and 2), and only one mismatch was allowed in the 8-bp BID sequence. The amplicon sequencing library protocol, which is based on ligation, produces nonoriented fragments with ~50% of the sequences oriented in the forwards/reverse orientation and ~50% of the sequences in the reverse/forwards orientation.

### Gene capture by hybridization (CBH)

In addition to metabarcoding, gene capture by hybridization (CBH)^79^ allows the enrichment of a broad range of genetic diversity of a gene, including rare or novel sequences, without bias introduced by PCR amplification^80,81^. In addition, it leads to the reconstruction of the full-length genes required to obtain a robust phylogenetic position of known or, more importantly, apparently novel taxa. This approach is particularly interesting for complementing metabarcode data for samples where the data suggest the occurrence of rare or unknown biodiversity.

Unlike metabarcoding, which relies on PCR amplification of fragments of limited size, CBH uses probes that are designed to be complementary to the targeted genes of the taxa of interest and are scattered throughout the genes to isolate them before sequencing. The effectiveness of gene capture depends heavily on selecting the optimal probe set, considering the immense diversity of organisms for which DNA can be present in complex environmental samples. Our approach involved designing degenerate probes to capture both known and potentially unknown genetic diversity while maintaining the desired specificity. This strategy involves the use of the known genetic diversity of a gene to anticipate and capture novel sequences. Classically, CBH is implemented on sequencing libraries, and a subsequent step of bioinformatic gene reconstruction is often required to recover complete gene sequences.

During the project, a pilot experiment was performed to validate the effects of existing CBHs on the 16S rRNA gene (prokaryotes) from deep-sea sediment, and CBH protocols were used to capture a broad range of metazoans by reconstructing the COI and 18S rRNA genes.

During the pilot study^34^, CBH was successfully applied to the 16S (bacterial and archaeal diversity) and 18S (metazoan diversity) genes, but the COI first trial failed to deliver the expected results (unpublished data gathered during the study by Gunther *et al*.^34^). The path for improving the 18S rRNA gene included optimizing the sequencing depth to better recover the full diversity of metazoans on the basis of full-length barcodes. For the COI gene, enrichment failed (personal communication) for seemingly more complex reasons. The lower abundance of COI genes and the use of a probe panel designed only on a subset of COI gene diversity (few full-length COI gene sequences were available at the time of probe design) are likely factors responsible for the suboptimal performance of CBH for COI enrichment. Probe design to target more comprehensive and diverse COI gene variants is now available, and optimizing the CBH protocol and conducting pilot studies with controlled samples are potential strategies to increase the efficacy of CBH for COI enrichment.

CBH experiments were conducted as described previously^82,83^, with the protocols used and developed in the framework of the “Pourquoi *Pas les Abysses*? The project is detailed below.

#### Probe design

##### Probes designed to target 16S and 18S rRNA Genes

Fifteen 16S rRNA gene-specific probes were previously designed^79^ to target the overall known bacterial and archaeal diversity (Table 3). The applied methodology was replicated to design probes targeting the 18S rRNA gene^34^. A rigorously curated database encompassing the full diversity of known 18S rRNA genes was compiled from the EMBL database^80,84^. Probes were designed via KASpOD and PhylArray^85^ software, which allows the determination of degenerate exploratory probes. Every k-mer ranging from 31 to 50 nucleotides in length was extracted from the reference sequences. Fully overlapping k-mers were clustered at an 88% identity threshold to produce a degenerate consensus sequence that reflects sequence variability at each nucleotide position. Some of the combinations derived from each degenerate probe potentially correspond to sequence variants that were previously absent from public databases. Probes were selected on the basis of their high coverage and their uniform distribution throughout the entire length of genes. A total of 17 probes were designed to target the 18S rRNA gene (Table 3). To facilitate large-scale probe production in a cost-effective manner, adaptor sequences were added to the ends of the probes to allow their amplification via PCR. The resulting sequence was “ATCGCACCAGCGTGT-NX-CACTGCGGCTCCTCA”, where “NX” represents the gene-specific probe.

##### Design of capture probes targeting the COI gene

An early MIDORI database^86^ containing 20 505 COI gene sequences from metazoans was used for the design of COI-specific probes. Probes were designed via KASpOD software, which employs a methodology similar to that used for 16S and 18S rRNA gene probes and uses a k-mer length of 30 nucleotides. Finally, a total of 13 probes were designed to specifically target the COI gene (Table 3).

#### Synthesis of biotinylated RNA probes

First, the designed probes were PCR-amplified to generate double-stranded DNA probes. For each probe, one PCR was carried out. The PCR mixtures (50 µL final volume) contained 0.2 µM final concentration of probe, 0.2 mM final concentration of dNTP mixture, 2 mM final concentration of MgSO_4_, 0.2 µM final concentration of primer T7-A 5′-GGATTCTAATACGACTCACTATAGGGATCGCACCAGCGTGT-3′ and primer B 5′-CGTGGATGAGGAGCCGCAGTG-3′, 1 U of Platinum *Taq* DNA Polymerase High Fidelity (Thermo Fisher Scientific) and 1 × final concentration High Fidelity PCR Buffer. A negative PCR control was carried out with nuclease-free water instead of the probe. The cycling conditions included a 2 min denaturation step at 94 °C followed by 35 cycles of 30 s at 94 °C, 30 s at 58 °C and 20 s at 68 °C and a final elongation step at 68 °C for 5 min. Probe amplification was checked with a 5 µL PCR aliquot by electrophoresis on a 2% agarose-TBE gel containing 0.5 × SYBR Safe DNA gel stain (Thermo Fisher Scientific). When a single band at the expected size was observed, the remaining 45 µL of amplified products was purified with the MinElute PCR Purification Kit (Qiagen) and eluted in 30 µL of nuclease-free water. When two amplification bands were observed, the remaining 45 µL of PCR products were purified from the agarose gel via the MinElute Gel Extraction Kit (Qiagen) and eluted in 20 µL of nuclease-free water. The concentration of the purified DNA double-stranded probes was measured with a Nanodrop spectrophotometer (Thermo Fisher Scientific).

Biotinylated RNA probes were subsequently synthesized. For this purpose, an equimolar solution of all amplified double-stranded DNA probes specific to one gene (16 rRNA genes, 18S rRNA genes or COI genes) was prepared, accounting for the degeneracy and length of each probe. This ensures that each probe combination is present in the same molecular amount. *In vitro* transcription (IVT) was performed with the MEGAscript T7 Transcription Kit (Ambion; Thermo Fisher Scientific), and Biotin-16-UTP (Roche Diagnostics; Sigma Aldrich, Mannheim, Germany) was used to produce biotinylated RNA probes. Each IVT reaction (20 µL final volume) contained 150 ng of a previously prepared mix of DNA double-stranded probes, a 7.5 mM final concentration of ATP solution, a 7.5 mM final concentration of CTP solution, a 7.5 mM final concentration of GTP solution, a 5.6 mM final concentration of UTP solution, a 1.9 mM final concentration of biotin-16-UTP, 1 × reaction buffer and 2 µL of T7 enzyme mixture (number of units not available). The IVT mixture was incubated at 37 °C for at least 6 hours. Two U of TURBO DNase was added to each IVT reaction and incubated at 37 °C for 30 min. IVT reactions were adjusted to 100 µL by adding 80 µL of nuclease-free water and dividing it into 2 equal volumes. Biotinylated RNA probe mixtures were purified with the RNeasy Plus Mini Kit (Qiagen). The purified probe mixtures were eluted in 40 µL of TE buffer. The concentration of the purified biotinylated RNA probe mixture was measured on a Qubit 3.0 fluorometer via a Qubit RNA HS assay kit. The average RNA probe mix length was checked via capillary electrophoresis on an Agilent TapeStation 4150 instrument via an RNA ScreenTape system. The RNA probe mixtures were stored at −80 °C until use.

#### Library construction

Libraries with a 550 bp insert size were prepared for the 10 samples of the CBH pilot study. For one sample (1 A), owing to low DNA quantity, a library was prepared using 1 ng of genomic DNA following the procedure of the Nextera XT DNA sample preparation kit (Illumina) (Illumina Reference guide #15031942 v02, April 2017). For the other nine samples, libraries were prepared via the TruSeq DNA Nano (Illumina) protocol according to the Illumina procedure (Illumina Reference guide #1000000040135v00, October 2017) with some modifications. Between 52 and 99 ng was fragmented via the Bioruptor system (Diagenode, Ougrée, Belgium) with 5 cycles of 15 sec “on” and 90 sec “off”. Fragmented DNA was purified with 1.6 × volumes of SPB beads (provided within the Illumina kit) and resuspended in 30 µL of RSB buffer (provided within the Illumina kit). Fragments were end-repaired with 20 µL of ERM2 reagent (provided within the Illumina kit) and size-selected via 80 µL of a mixture of 1:1 PCR-grade water:SPB beads to remove large DNA fragments and 15 µl of undiluted SPB beads mixed with 125 µl of the obtained supernatant to remove small fragments. Size-selected fragments were resuspended in 17.5 µL of RSB buffer before proceeding with 3′ A-tailing, adapter ligation and PCR amplification (8 cycles) following the Illumina procedure.

#### Library quality control

All the libraries were quantified via a Quant-iT dsDNA HS Assay using a Qubit 3.0 Fluorometer instrument (Thermo Scientific). Library profiles were assessed via capillary electrophoresis on an Agilent Bioanalyzer via a high-sensitivity DNA kit (Agilent Technologies).

#### Library amplification

Library amplification was performed via the GC-RICH PCR System dNTPack Kit (Roche Applied Science, Basel, Switzerland). Library volumes were adjusted to 50 µL. Between 3 and 5 PCRs were performed per library via primers fully complementary to Illumina adapters to obtain a minimum of 500 ng of each library. Each amplification reaction (final volume of 50 µL) contained 5 µL of the prepared library, 1 mM MgCl_2_, 0.2 mM final concentration of PCR Grade Nucleotide Mix, 0.5 µM final concentration of TS-PCR Oligo 1 5′-AATGATACGGCGACCACCGAGA-3′, 0.5 µM final concentration of TS-PCR Oligo 2 5′-CAAGCAGAAGACGGCATACGAG-3′, 1 × final concentration of GC-RICH PCR buffer and 2 U of GC-RICH Enzyme Mix. The cycling conditions included a 4 min denaturation step at 94 °C; 20 cycles of 30 s at 94 °C, 1 min at 58 °C and 1 min 30 s at 68 °C; and a final elongation step at 68 °C for 3 min. PCR-amplified libraries were pooled into 2.5–3 reactions, cleaned with a 0.6 × volume of AMPure XP beads (Beckmann Coulter) and resuspended in 50 μL of nuclease-free water. The profile of each purified amplified library was assessed via capillary electrophoresis via an Agilent TapeStation 4150 instrument and the D5000 ScreenTape system. Libraries were quantified on a Qubit 3.0 fluorometer instrument via a Qubit DNA HS assay kit. Purified-amplified libraries were stored at −20 °C.

#### Hybridization experiment

Hybridization was performed independently for each library. Five hundred nanograms of purified amplified library were mixed with 2.5 μg of sheared salmon sperm DNA (Invitrogen; Thermo Fisher Scientific) and incubated with 500 ng of biotinylated RNA probe mixture (mixed in a 50:50 ratio for 16S and 18S probe sets, alone for the COI probe set) and 1 × final concentration of hybridization buffer (containing 10 × SSPE, 10 × Denhardt’s solution, 10 mM EDTA, 0.2% SDS) for 24 h at 65 °C. Hybridized probe/target complexes were captured via 500 μg of Dynabeads M-280 Streptavidin, which are paramagnetic streptavidin-coated beads (Invitrogen; Thermo Fisher Scientific). The beads were collected via a magnetic stand, washed once with 500 μL of 1 × SSC/0.1% SDS buffer, and then three times with 500 μL of 0.1 × SSC/0.1% SDS buffer preheated at 65 °C. The captured libraries were eluted with 50 μL of 0.1 M NaOH. The eluted DNA was neutralized with 70 μL of 1 M Tris-HCl pH 7.5 buffer. The captured libraries were amplified via 25 cycles of PCR instead of 20 cycles and then purified as described in the previous section for library amplification. To further increase enrichment efficiency, a second round of capture was conducted as detailed in this paragraph.

#### Illumina sequencing

The quality-checked libraries were subjected to qPCR quantification via the KAPA Library Quantification Kit for Illumina Libraries (KapaBiosystems, Wilmington, MA, USA) on a CFX96 Real-Time PCR instrument (Bio-Rad, Hercules, CA, USA). These quantification results were employed to prepare stock solutions for equimolar mixing of libraries. Libraries enriched for the 16S/18S rRNA genes and COI gene were sequenced simultaneously on an Illumina MiSeq run (2 × 300 bp).

### Data quality control and filtering

#### Metagenomic sequencing quality control

As described by Alberti *et al*.^72^, for quality control, the reads that passed the Illumina quality filters were subjected to the following pruning and removal steps via in-house fastx_clean software (Code availability 4 & 5):

- Illumina sequencing of adaptors and primer sequences, as well as low-quality (Q < 20) nucleotides from read ends (the longest sequence free of adaptors and low-quality bases was retained).
- A second unknown nucleotide (N) remains until the end of the read.
- Reads shorter than 30 nucleotides (after trimming).
- In the final step, reads were mapped to the Enterobacteria phage PhiX174 genome (GenBank: NC_001422.1) and removed via Bowtie287 v2.2.9 (-L 31 --mp 4 --rdg 6,6 --local --no-unal).

In addition, quality checks were performed on random subsets of 20,000 reads before (‘raw’ reads) and after the filtering steps (‘clean’ reads): (i) the rate of duplicated sequences was estimated from single- and double-ended raw sequences via fastx_estimate_duplicate (Code availability 7); (ii) read size, quality values, indeterminate base positions and base composition were calculated, and sequencing adapters were detected before and after read filtering; and (iii) taxonomic assignment was performed via Centrifuge v1.0.388 and the NCBI nonredundant nucleotide database.

#### Metabarcoding sequencing quality control

No quality trimming or adaptor trimming was performed on the metabarcoding sequencing data before the metabarcoding sequencing data were not quality controlled or adapter adjusted prior to application of the eDNAbyss pipeline. However, quality control was performed on a random subset of 20,000 raw sequencing reads as described in the previous section, except that taxonomic assignment was performed via the SortMeRNA v2.189 and SILVA90 (v119) databases (for 16S and 18S experiments), the PR291 (v4.3.0) database (for 18S experiments) and the COI database (options: --best 1 --fastx --blast ‘1 cigar qcov’ --aligned rRNA -other not_rRNA --log -v --otu_map --de_novo_otu --id 0.97 --coverage 0.97). The COI database was composed of COIs downloaded from NCBI. This taxonomic assignment project enabled us to estimate sample quality and avoid sample inversions. Negative controls (NCs) for extraction and PCR were used for the metabarcoding experiments. As described in Belser *et al*.^72^, taxonomic assignment was carried out to track possible contaminating species that may be detected in the reagents and to build a database. Briefly, the NC sequences were adapted and cleaned with fastx_clean. The sequences were then merged with USEARCH^87^ v9.2.64 (Code availability **9**) (-fastq_mergepairs *.fastq -fastqout merged.fq -relabel @ options). The merged sequences were quality-filtered (-fastq_filter merged.fastq -fastq_maxee 1.0 -fastaout merged.fa -relabel options), dereplicated (-derep_fulllength merged.fa -sizeout -relabel Uniq -fastaout merged.uniques.fa options), and clustered (-cluster_otus merged.uniques.fa -minsize 2 -otus otus.fa -relabel Cluster options). Taxonomic assignment of clusters was then performed with SortMeRNA (options: --best 1 --fastx --blast ‘1 cigar qcov’ --aligned rRNA -other not_rRNA --log -v --otu_map --de_novo_otu --id 0.97 --coverage 0.97) and the SILVA databases^88^ (Code availability **6**), the PR2 database^89^ and the COI database for the SSU, 18S and COI experiments, respectively. The cluster abundance was calculated via USEARCH^87^, and a table of operational taxonomic units (OTUs) was generated (-usearch_global *.fq -db assigned_clusters.fa -strand plus -id 0.97 -log make_otutab.log -otutabout otutab.txt options). A database for each NC sample was created from the clusters. Finally, the sequences of each sample were compared via SortMe RNA to their relative NC databases to provide a first appraisal of the amount of possible contamination caused by the reagents.

#### CBH quality control

Following a previously published analysis protocol^82^, the raw sequencing reads were first trimmed for Illumina adapters via Trimmomatic^90^ v0.38. Subsequently, quality trimming and filtering were performed via the PRINSEQ-lite PERL script v0.20.4, with the following parameters: min_qual_mean = 25, trim_qual_window = 3, trim_qual_step = 1, and the exclusion of reads shorter than 60 bp after trimming (min_len = 60). A read quality assessment was performed before and after trimming via FastQC^91^. Alternatively, the newly available metagenomic classifier RiboTaxa, which includes quality control and filtering, can be employed (see 4.3 CBH pipeline).

## Data Records

Basic metadata are registered in BioSamples (as detailed in the section on association of metadata) and available in open access since their registration, prior to sequencing (https://www.ebi.ac.uk/biosamples/samples?text=eDNAbyss) in accordance with the FAIR principles for data and metadata^92,93^. A tabular version of the full sample registry, including a description of their metadata fields, is available via open access on Zenodo https://zenodo.org/records/6815677)^94^. Sample provenance and environmental context were documented via a combination of controlled fields from community checklists such as GSC MIxS water (ERC000024) and ENA MicroB3 (ERC000027) and proprietary fields that further enhanced “FAIRness”.

All sequencing files are available (in the usual fastq.gz format) in the European Nucleotide Archive (ENA) at the EMBL European Bioinformatics Institute (EMBL-EBI) under the eDNAbyss Umbrella BioProject (https://www.ebi.ac.uk/ena/browser/view/PRJEB3922537). Each manuscript is associated with a nested bioproject. Table 4 includes the architecture of the projects at ENA with the detailed code and content of each nested bioproject. A detail of all information associated to data at ENA is listed in the Table S2.

## Technical Validation

### Sample and information management (NGL)

An internal LIMS called NGL (next-generation LIMS) named NGL (Next Generation LIMS) was created by Belser *et al*.^73^ to handle the extensive data generated for each sample and ensure that these data remain connected and traceable throughout the workflow. Briefly, as detailed in the “Technical Validation”, “Sample and experiments information management” of this article, NGL links sample metadata from the moment of collection through to the deposition of sequencing files at EMBL-EBI, thereby enabling storage, monitoring, and retrieval of information at every step, as well as statistical reporting and troubleshooting. NGL is organized into specialized modules with NGL-P that manages projects and NGL-SQ (SeQuencing), tracking input material, reagents, sequencing output, and QC steps. A web interface enables batch data entry and monitoring of experiments up to sequencing.

After sequencing, run information is stored in NGL-BI (Bioinformatics), which interfaces with NGS-QC pipelines via a REST API to perform analyses. QC metrics and plots are stored in NGL-BI and accessible online for validation of processed files. For metabarcoding, a dedicated QC pipeline was applied, and comparisons with negative controls were carried out using the NGS-BA (Biological Analysis) module.

Eventually, the workflow concludes with NGL-SUB (Submission), which automates the deposition of cleaned sequencing data to the ENA (EMBL-EBI), ensuring that files are properly linked with their respective Biosamples and metadata.

### Sequencing quality control

Specific quality controls were implemented at each stage of sample processing from DNA extraction to library preparation, as detailed in the distinct sections above.

The sequencing quality process was similar to that described by Belser *et al*.^73^. Briefly, the following procedures were applied:

For metagenome data, all metadata (unique eDNAbyss identifier, taxonomic group, sampling site, and library type) together with the information generated during the QC workflow (here, only sequencing runs with more than 80% reads exhibiting Q-values > 30 were used) were recorded in the LIMS and could be accessed via the NGL-BI web interface (see Figure S6 in in Belser *et al*.^73^). This platform enables sequencing files to be validated based on their QC statistics. Multiple panels allow visualizing the number of reads obtained for each sequencing library to compare the distribution of base composition and Q-scores across the read length both before and after cleaning. Cleaning consists of trimming the read ends for Q-value < 10 and any remaining sequencing adapters, with only sequences > 35 bp kept after trimming. Post-cleaning statistics, such as the number of trimmed reads and nucleotides removed, are provided in a “Trimming” panel, to consider discarding files with a large fraction of discarded reads indicating poor sequencing quality. Taxonomic assignment results are also summarized in a dedicated panel, reporting the number of sequences classified and their taxonomic labels. It is common to observe a relatively large proportion of unassigned reads, particularly in planktonic samples originating from water filtration or sediments.

For metabarcoding libraries, as no cleaning is performed at this stage, only the QC workflow was used. Base composition often appears biased since only a very short and conserved genomic region is amplified. Statistics from the merging of paired reads are particularly informative, as the resulting fragment size must match the expected amplicon length for the targeted locus; otherwise the sample is discarded and the experiment is repeated. It should be noted here that in 16S and 18S assays, eukaryotic sequences may be recovered using 16S primers and, conversely, bacterial or archaeal sequences may be recovered 18S primers, which inevitably leads to bimodal length distributions.

Taxonomic classifications are displayed in the same manner as for metagenomic datasets. To identify potential contaminants introduced during the experiments, every sample was compared against three negative control (NC) databases — one from extraction and two from PCR — using SortMeRNA. The proportion of suspect sequences was calculated for each sample/control comparison, and a report generated. Candidate contaminant clusters are were manually curated, excluding sequences from likely non-contaminant taxa or poorly resolved assignments. Reads assigned to validated contaminant clusters are usually removed from the dataset; however, this was not necessary in any of the present project’s runs.

The sequences are eventually posted at ENA and made available alongside the associated post-sequencing metadata listed in Table S2.

### Bioinformatic analysis

#### A Flexible pipeline for metabarcoding

**Fig. 5:**
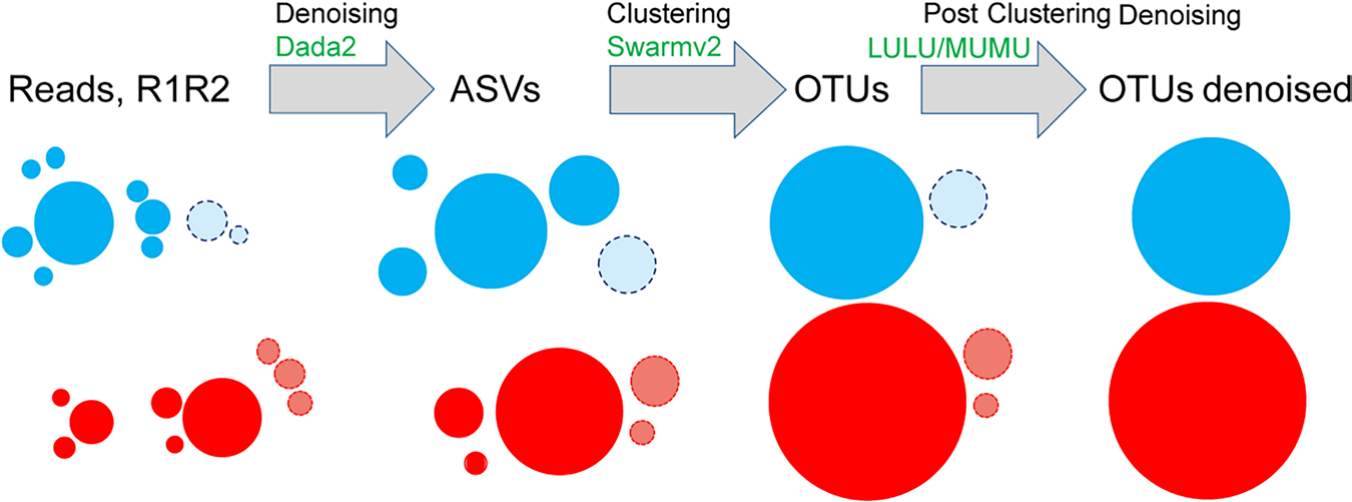
Scheme of the steps of the bioinformatic pipeline. These include the cleaning and assembly of raw paired-end reads (R1, R2), the denoising and delineation of sets of identical sequences into ASVs, the clustering of ASVs into OTUs, and their postclustering curation, which allows the removal of the remaining spurious OTUs (e.g., nontarget sequences, such as nuclear mitochondrial DNA [numts] and paralogues).

A flexible pipeline, available through GitLab (https://gitlab.ifremer.fr/abyss-project/), was designed to allow the combined analysis of metabarcode data for prokaryotes, unicellular eukaryotes and metazoans^35^, combining several existing algorithms (Fig. 5). The parameters are informed on GitLab and updated as the tools evolve. For example, further refinements of this pipeline were performed as part of a latter project (*MarEEE*, dedicated to marine biodiversity and nonindigenous species in coastal waters) to format the taxonomic output of the data and to include improved algorithms (see below).

First, a standard denoising step *via* the DADA2 pipeline^95^ was used to correct for sequencing errors and generate tables of amplicon sequence variants (ASVs). From this step, the DADA2 pipeline allows the merging of ASV tables obtained with the same barcode but originating from distinct sequencing runs. ASV tables are then checked and cleaned for contaminants via the package *decontam* (https://github.com/benjjneb/decontam). A clustering step using Swarm^96^ and a postclustering curation step using the MUMU algorithm^97^ developed from LULU^98^ were also included, although they are optional steps, depending on the primers used, the organisms targeted and the questions to be addressed. Clustering allows the formation of groups of similar ASVs (i.e., operational taxonomic units [OTUs] or molecular taxonomic units [MOTUS]) that are the closest analogues to metazoan species for taxonomic and ecological surveys^99^.

ASV tables are essential resources that will ultimately be made publicly available, associated with the metadata shared in Zenodo^94^, since they are a key step needed for future combinations of results from distinct studies. ASVs are essential for moving towards a global appraisal of biodiversity through the combination of results from multiple independent studies. Compared with OTUs, ASV tables also allow changes in taxonomy, which is a dynamic field, particularly in deep-sea environments, and updates of the reference database (e.g., discoveries of new lineages reducing the uncertainties/errors in ASV clustering). Many recommendations have been made to favour the sharing of ASVs (rather than OTUs) in databases, starting with Callahan *et al*.^100^ This is mostly due to interest in the information they deliver about genetic diversity within lineages (and thus OTUs) and, as explained above, their cross-compatibility (for merging datasets) and comparability across studies. We, however, wish to emphasize that although only the ASV table is shared, as it is the essential step in allowing further incremental additions of data, this is not necessarily the final step in the analysis. Depending on the organisms and ecosystems targeted and the scientific questions addressed, clustering ASVs into OTUs remains relevant^35,101^, as grouping ASVs on the basis of their taxonomic assignments is theoretically sufficient at the condition reference databases allow accurate taxonomic assignment at lower taxonomic levels (e.g., species or genera), a situation is at odds with marine biodiversity, let alone the deep sea, where reference databases are not comprehensive^27,35^ or devoid of errors, a long-recognized^102^ and yet unresolved problem^103^.

#### Metagenome assembly

All analyses of the demultiplexed metagenomic reads started with quality filtration via Illumina-util python scripts^104^, following the recommendations of Minoche *et al*.^105^ The following steps in genome reconstruction were carried out through a publicly available snakemake workflow integrating standard metagenomic tools with Anvi’o’s analysis and visualization platform^106–108^. Briefly, the workflow carries out the following steps: (i) metagenomic assembly, either individually or by groups of samples (i.e., coassembly), (ii) read recruitment of the raw reads to the assembled contigs, (iii) characterization of the contigs (gene prediction, functional and taxonomic assignment), and (iv) automatic binning with a variety of algorithms available (e.g., CONCOCT^109^ or Metabat2^110^). Following this automatic grouping of contigs into bins, on the basis of tetranucleotide frequency and differential coverage, they can be manually refined at Anvi’o’s interface or automatically refined with DASTool^111^ before being dereplicated to obtain a collection of nonredundant metagenome-assembled genomes (MAGs). The completeness and redundancy of the MAGs were estimated with CheckM^112^, and a new read recruitment step of all the samples against the MAGs yielded an observation table, similar to an ASV table. Taxonomic annotation of the MAGs can be obtained from CheckM or by phylogenomic placement in the GTDB database^113^ via GTDB-tk^114^. Two cases in which these methods were used to reconstruct MAGs (see bioproject PRJEB60556 in Table 4) from eDNAbyss samples were described by Trouche *et al*.^44^ and Schauberger *et al*.^45^

#### CBH Pipeline

CBH theoretically allows the recovery of complete rDNA sequences by employing probes distributed throughout the whole length of small subunit (SSU) rRNA genes. As CBH is typically performed on fragmented sequences generated during Illumina library preparation, a subsequent assembly step is often required to reconstruct full-length sequences of the targeted genes.

#### Bioinformatic analysis of 16S/18S rRNA Gene CBH sequencing data

We exploited reference-guided assemblers to reconstruct near-full-length to full-length 16S and 18S rRNA sequences that allowed the reconstruction of distant variants while effectively preventing chimaera formation. To mitigate the potential issue of chimaeras present in reference databases, we employed the most current and well-curated 16S/18S rRNA gene database, SILVA SSURef NR99^88^ (release 132).

CBH sequencing data were analysed following a previously published analysis protocol^82^ involving SSU read filtering with SortMeRNA v2.1 and SSU rDNA gene reconstruction via EMIRGE v0.61. For taxonomic classification of reconstructed SSU rDNA gene sequences, the most up-to-date plugin “feature-classifier sklearn classifier”^115^ from QIIME2 v2019.1 was employed instead of the script assign_taxonomy.sh from QIIME1. For each step, SSURef NR99^88^ (release 132) was used as a reference database.

**Fig. 6:**
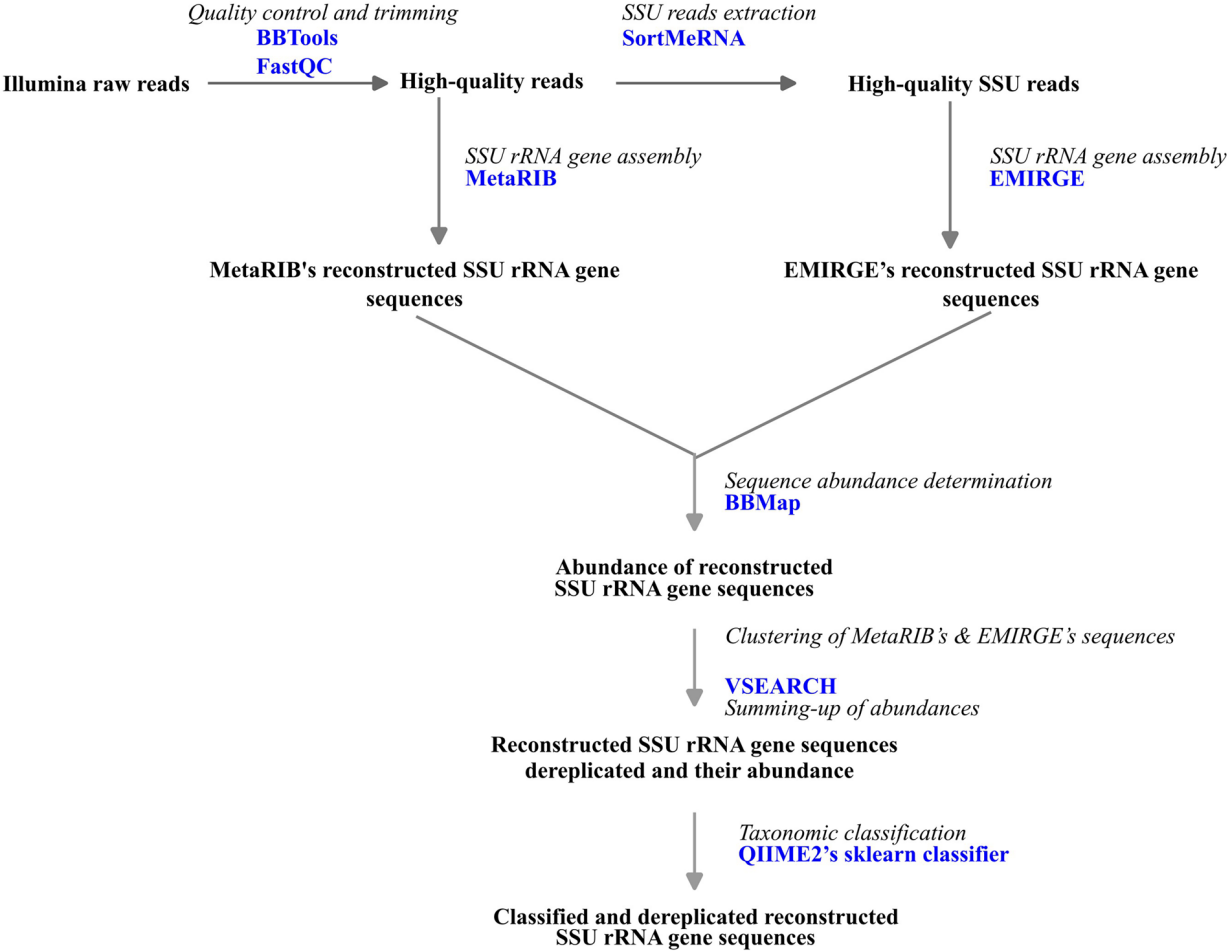
Schematic of the bioinformatic pipeline steps used to recover prokaryotic and eukaryotic diversity from CBH sequencing data. The pipeline included raw read quality assessment, adapter removal and trimming, 16S/18S rDNA rDNA read filtering, reconstruction of full-length SSU rRNA sequences, and classification of full-length reconstructed SSU sequences.

Building on this analysis pipeline, a metagenomic classifier named RiboTaxa^116^ was developed to increase diversity recovery. RiboTaxa is a comprehensive toolset that handles raw read quality assessment, adapter removal and trimming, 16S/18S rDNA read filtering, reconstruction of full-length SSU rRNA sequences, and classification of full-length reconstructed SSU sequences (Fig. 6). Tools compiled in this pipeline were selected by benchmarking the performance and accuracy of rRNA-specialized versus general-purpose read mappers, reference-targeted assemblers and taxonomic classifiers.

The initial step involved removing Illumina adapters and trimming reads via *bbduk.sh* (https://sourceforge.net/projects/bbmap/). Quality assessment was performed before and after trimming with FastQC (https://github.com/s-andrews/FastQC), and summary statistics were compiled in a standalone HTML file via MultiQC^117^. For reconstructing SSU rRNA genes, RiboTaxa utilizes two SSU rRNA gene reference-based assemblers, MetaRib^118^ (https://github.com/yxxue/MetaRib) and EMIRGE^119^ (Emirge_amplicon.py). The combined use of these two assemblers has been shown to improve microbial diversity^116^. MetaRib employs unfiltered high-quality reads for SSU rRNA gene assembly, whereas EMIRGE utilizes SSU reads extracted through SortMeRNA^120^. Once the full-length SSU rRNA gene sequences have been reconstructed, their relative abundances are determined by mapping unfiltered high-quality reads against them via BBmap^121^. The full-length SSU rRNA gene sequences generated by MetaRib and EMIRGE were subsequently clustered at a 97% sequence identity threshold via VSEARCH. This step is used to reduce sequence redundancy obtained from the two assemblers. The sequence abundances obtained from BBmap were then summed for sequences belonging to the same cluster. Finally, the clustered sequences were taxonomically classified via QIIME 2’s classify-sklearn plugin^115^. Given that RiboTaxa integrates multiple tools, the essential parameters for each tool (bbduk.sh, EMIRGE, MetaRib, and sklearn_classifier) are defined in the RiboTaxa_arguments.conf file, which provides detailed explanations for each setting. Thread and memory allocation can also be adjusted by the user. RiboTaxa and all the parameter recommendations are available at GitHub https://github.com/oschakoory/RiboTaxa. The nonredundant SILVA SSU database is the preferred database for SSU read filtering via SortMeRNA, SSU rRNA gene reconstruction, and taxonomic assignment.

Additionally, a Kraken2-based^122^ analysis was conducted using paired-end reads to assess the full range of captured diversity without reconstructing genes. This approach was employed to prevent low-coverage taxa from hindering the reconstruction of longer sequences, which could lead to their exclusion from the final dataset. Kraken2 analysis was performed with the prepackaged SILVA database. To determine the optimal confidence score, we evaluated values ranging from 0.0 to 1.0 in increments of 0.1. For the final analyses, a minimum score of 0.7 was selected, ensuring specificity in the taxonomic assignments. This decision aligns with a previous study^123^ that indicated that confidence scores between 0.6 and 0.7 produced the best trade-off between sensitivity and precision.

#### Bioinformatic analysis of COI Gene CBH sequencing data

A *de novo* assembly approach was implemented to recover full-length COI gene sequences from CBH sequencing data. Initially, trimmed reads were filtered to eliminate SSU rRNA reads via SortMeRNA v2.1 with the SILVA database (release 132). Non-SSU rRNA reads were assembled via IDBA-UD^124^ with the parameters mink = 10, maxk = 120, step = 10, sim = 0.99, and precorrection, and the resulting contigs were subsequently extended via CAP3^125^. Contigs corresponding to COI genes were then identified through tBLASTx (all genetic codes were tested) against the MIDORI COI database (version GenBank237, including the longest sequences).

## Supplementary information

Supplementary information

## Data Availability

The global dataset has been deposited in European Nucleotide Archive (ENA) as project PRJEB 39225 (https://www.ebi.ac.uk/ena/browser/view/PRJEB39225), with metadata available on Zenodo (https://zenodo.org/records/6815677).

## Code availability

1. Time Analysis software: https://www.illumina.com/search.html?filter=support&q=RTA%20download&p=1.

2. Bcl2fastq Conversion: https://support.illumina.com/downloads/bcl2fastq-conversion-software-v2-20.html

3. Cutadapt, https://github.com/marcelm/cutadapt/releases/tag/v1.18

4. Fastx_clean software, http://www.genoscope.cns.fr/fastxtend

5. FASTX-Toolkit, http://hannonlab.cshl.edu/fastx_toolkit/index.html

6. SortMeRNA v2.1, https://github.com/biocore/sortmerna

7. fastx_estimate_duplicate software, http://www.genoscope.cns.fr/fastxtend

8. fastx_mergepairs software, http://www.genoscope.cns.fr/fastxtend

9. Usearch, https://www.drive5.com/usearch/

10. Trimmomatic: https://github.com/usadellab/Trimmomatic

11. Decontam: https://github.com/benjjneb/decontam

12. Prinseq: https://github.com/uwb-linux/prinseq

13. Qiime2 feature classifier: https://github.com/qiime2/q2-feature-classifier

14. FastQC: https://github.com/s-andrews/FastQC

15. BBTools: https://github.com/kbaseapps/BBTools

16. MultiQC: https://github.com/MultiQC/MultiQC

17. MetaRib: https://github.com/yxxue/MetaRib

18. EMIRGE: https://github.com/csmiller/EMIRGE

19. VSearch: https://github.com/torognes/vsearch

20. 1IDBA_UD: https://github.com/1928d/idba_ud

21. CAP3: https://faculty.sites.iastate.edu/xqhuang/cap3-and-pcap-sequence-and-genome-assemblyprograms

22. eDNAbyss pipeline: https://gitlab.ifremer.fr/abyss-project/

23. MUMU algorithm: https://github.com/frederic-mahe/mumu

24. bbmap: https://sourceforge.net/projects/bbmap/

26. RiboTaxa: https://github.com/oschakoory/RiboTaxa

27. eDNAbyss pipeline(s): https://gitlab.ifremer.fr/abyss-project/),
