# Supplementary material for: Omics exploration of deep-sea biodiversity: data from the “Pourquoi Pas les Abysses?” and eDNAbyss projects
Source: Sci Data. 2025 Dec 20;12:1982. doi: 10.1038/s41597-025-06009-1 (PMC12727693; doi:10.1038/s41597-025-06009-1)
Supplement: Supplementary file 1 — Supplementary information [file 41597_2025_6009_MOESM1_ESM.docx]

**Supplementary File 1. Tables**

**Supplementary Table S1.** List of metadata, with their units and sources collected during the cruises or obtained through the analysis of sediment samples and the use of worldwide databases.

| **Variable** | **Unité** | **Source** |
| --- | --- | --- |
|  |  |  |
| GPS coordinates | DD | Field acquisition |
| Depth | m | Field acquisition |
| Date | DD/MM/YY | Field acquisition |
| Name of the ship | °C | Field acquisition |
| Name & doi of the cruise | °C | Field acquisition |
| Marine region code | °C | World Marine Region |
| Biome | °C | ENVO |
| Habitat feature | °C | ENVO |
| Distance to the nearest coast | °C | R & naturalearthdata.com |
| Temperature (mean & SD) | °C | Bio-Oracle v2.1/ARMOR |
| Salinity (mean & SD) | PSS | Bio-Oracle v2.0/ARMOR |
| Current velocity (mean & SD) | m.s^-1^ | Bio-Oracle v2.1/ORAP |
| Dissolved oxygen (mean & SD) | mol.m^-3^ | Bio-Oracle v2.1/PISCES* |
| Nitrate (mean & SD) | mol.m^-3^ | Bio-Oracle v2.1/PISCES |
| Iron (mean & SD) | umol.m^-3^ | Bio-Oracle v2.1/PISCES |
| Silicate (mean & SD) | mol.m^-3^ | Bio-Oracle v2.1/PISCES |
| Chlorophyll a (mean & SD) | mg.m^-3^ | Bio-Oracle v2.1/PISCES |
| Phosphate (mean & SD) | mol.m^-3^ | Bio-Oracle v2.1/PISCES |
| Phytoplanctonic carbon (mean & SD) | umol.m^-3^ | Bio-Oracle v2.1/PISCES |
| Surface primary productivity (mean & SD) | g.m-3.day^-1^ | Bio-Oracle v2.1/PISCES |
| Sediment water content (mean & SD) | % | Samples analysis |
| Sediment organic matter content (mean & SD) | % | Samples analysis |
| Sediment Granulometry (Dv_span_ (mean & SD) | µm | Samples analysis |
| Region | Code | ENVO |
| Biome | Code | ENVO |
| Sediment type | (Supp. File 2) | SHOM 2011 World Sedimentary Chart v7.1 |

## **Supplementary Table S2 :** Detail of the file and variable available at ENA with this dataset, which can be viewed by following the drop-down menu « Show column selection ».

| bam_bytes | Size (in bytes) of BAM files |
| --- | --- |
| bam_ftp | FTP links for BAM files |
| bam_md5 | MD5 checksum of BAM files |
| base_count | Number of base pairs |
| broker_name | Name of broker for the submission |
| center_name | Submitting centre |
| experiment_accession | Experiment accession number |
| experiment_alias | Submitter’s name for the experiment |
| experiment_title | Brief experiment title |
| fastq_aspera | Aspera links for FASTQ files |
| fastq_bytes | Size (in bytes) of FASTQ files |
| fastq_ftp | FTP links for FASTQ files |
| fastq_galaxy | Galaxy links for FASTQ files |
| fastq_md5 | MD5 checksum of FASTQ files |
| first_created | Date when first created |
| first_public | Date when made public |
| instrument_model | Instrument model used in sequencing experiment |
| instrument_platform | Instrument platform used in sequencing experiment |
| last_updated | Date when record was last updated |
| library_layout | Sequencing library layout |
| library_name | Sequencing library name |
| library_selection | Method used to select or enrich the material being sequenced |
| library_source | Source material being sequenced |
| library_strategy | Sequencing technique intended for the library |
| nominal_length | Average fragmentation size of paired reads |
| read_count | Number of reads |
| run_accession | Run accession number |
| run_alias | Submitter’s name for the run |
| sample_accession | Sample accession number |
| sample_alias | Submitter’s name for the sample |
| sample_title | Brief sample title |
| scientific_name | Scientific name of the organism from which the sample was derived |
| secondary_sample_accession | Secondary sample accession number |
| secondary_study_accession | Secondary study accession number |
| sra_aspera | Aspera links for NCBI SRA format files |
| sra_bytes | Size (in bytes) of NCBI SRA format files |
| sra_ftp | FTP links for NCBI SRA format files |
| sra_galaxy | Galaxy links for NCBI SRA format files |
| sra_md5 | MD5 checksum of NCBI SRA format files |
| study_accession | Study accession name |
| study_alias | Submitter’s name for the study |
| study_title | Brief sequencing study description |
| submission_accession | Submission accession number |
| submitted_aspera | Aspera links for submitted files |
| submitted_bytes | Size (in bytes) of submitted files |
| submitted_format | Format of submitted reads |
| submitted_ftp | FTP links for submitted files |
| submitted_galaxy | Galaxy links for submitted files |
| submitted_md5 | MD5 checksum of submitted files |
| tax_id | NCBI taxon ID of the organism from which the sample was obtained |

**Supplementary File 2.** Metadata from the SHOM database.


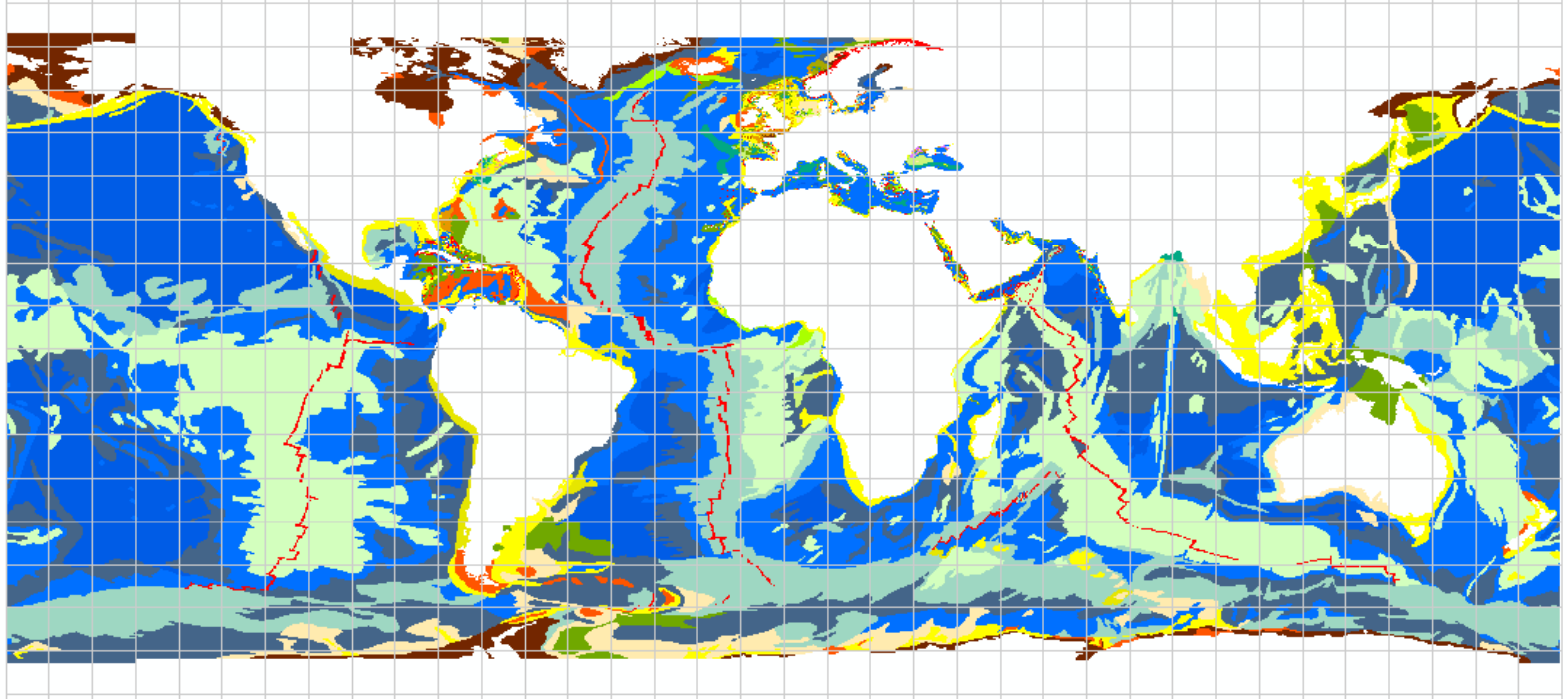


**Supplementary Figure S1.** Map of the categories of substrate derived from the SHOM database,

**Supplementary Table S3.** Subtrate Type and NF Classifications

| Substrate Type | NF Codes |
| --- | --- |
| Rock (Bedrock) | NFRoche, Roche, NFG |
| Gravel | NFS |
| Sand | NFSFC |
| Fine Sand | NFV |
| Mud (Vases) | NFC |
| Pebbles / Mixed Coarse Sediments | NFGC, NFCG, NFCS, NFCV, NFSV, NFSC, NFGV, NFSG, NFSGV, NFSFV |
| Silt | NFSi, NFSiA, NFSSi, NFVG |
| Clay | NFA, NFASi, NFVS, NFVSF |

**Definitions**

Sediment: A collection of detrital or biogenic particles of varying coarseness that have been transported by one or more transport agents.

NF: Nature of the bottom (“Nature du fond”).

C: Pebble: Sediment containing 50 to 100% particles larger than 20 mm. CG: Pebble-gravel: Sediment containing pebbles and 15 to 50% gravel.

G: Gravel: Sediment containing 50 to 100% particles between 20 and 2 millimetres. SG: Sand-gravel: Sediment containing sand and 15 to 50% gravel.

S: Sand: Sediment containing 50 to 100% particles between 2 and 0.5 millimetres.

SV: Silty sands: Sands containing between 5 and 20% particles smaller than 0.05 millimetres.

SF: Fine sands: Sediment containing 50 to 100% of particles between 0.5 and 0.05 millimetres.

SFV: Fine muddy sands: Fine sands containing between 5 and 20% particles smaller than 0.05 mm.

Si: Silts: Sediment containing 50 to 100% particles between 0.05 and 0.01 mm.

A: Clays: Sediment containing 50 to 100% particles smaller than 0.01 mm.

Vases: Sediment containing 20 to 100% particles smaller than 0.05 mm.
